# H_2_S‐Amplified “Three‐in‐One” Antibacterial Strategy for Periodontitis Treatment Using a Photosensitive Pillararene‐Embedded COF/MOF Hybrid

**DOI:** 10.1002/advs.75608

**Published:** 2026-05-07

**Authors:** Shuang Liang, Meng‐Hao Li, Liang Cheng, Tian‐Shou Zhang, Hui Hui, Yan Wang, Hong‐Pu Zhang, Bo‐Wen Liu, Lin Wang, Ying‐Wei Yang

**Affiliations:** ^1^ Department of Prosthodontics Jilin Provincial Key Laboratory of Sciences and Technology for Stomatology Nanoengineering School and Hospital of Stomatology Jilin University Changchun P. R. China; ^2^ College of Chemistry Jilin University Changchun P. R. China

**Keywords:** biofilm‐associated infections, covalent organic frameworks, multifunctional combination therapy, periodontitis, pillararene

## Abstract

Reactive oxygen species (ROS)‐based nanomedicine holds great promise for combating biofilm‐associated infections (BAIs). Nonetheless, the strong antioxidant systems in these microenvironments lessen the effectiveness of ROS. The combination of ROS generation and depletion of antioxidant pathways is a potential approach to this issue. Herein, we present COF/HKUST‐10, a composite platform of a copper‐based metal‐organic framework (HKUST‐1) and pillararene‐embedded covalent organic frameworks for BAIs. Both endogenous hydrogen sulfide and exogenous light activate this platform, thereby inducing synergistic catalytic reactions that enhance ROS generation and local antioxidant inhibition. Transcriptomic analysis revealed that ROS and Cu^+^/Cu^2+^ overload disrupted porphyrin metabolism in *Porphyromonas gingivalis*, severely impairing its energy metabolism and pathogenicity. This synergistic “three‐in‐one” antibacterial strategy involves ROS amplification, antioxidant system depletion, and copper ion‐mediated bactericidal effects. The experiments demonstrated the material's exceptional efficacy, resulting in robust antibacterial activity, efficient biofilm eradication, and significant reduction in inflammation. This study presents a distinctive strategy to enable synergistic treatment of BAIs, broadening the practical applications of supramolecular materials in biomedical applications.

## Graphical abstract

The integration of ROS‐generating systems with H_2_S depletion strategies effectively overcomes the limitations imposed by endogenous antioxidant defenses on ROS‐based antimicrobial therapies. In this study, the Cu‐MOF is incorporated into pillararene‐embedded COF, achieving a “three‐in‐one” antimicrobial effect that markedly alleviated periodontal inflammation. This work presents an innovative approach for the treatment of biofilm‐associated infections.

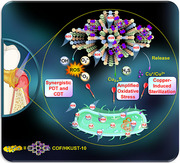

## 1 Introduction

Bacterial biofilm‐associated infections (BAIs) are persistent global health threats that exhibit drug resistance, persistent infection, and high recurrence [1, 2, 3, 4]. Modern nanomedical technologies that target biofilms via reactive oxygen species (ROS) for biofilm inhibition, such as photodynamic therapy (PDT), chemodynamic therapy (CDT), and sonodynamic therapy (SDT), are emerging research areas to effectively eradicate BAIs [5, 6, 7, 8]. These therapies employ externally generated ROS to induce oxidative stress, disrupting biofilm integrity and inactivating bacteria via nonspecific damage to biomolecules [9]. Hence, it is essential to develop advanced materials that effectively catalyze ROS generation to benefit next‐generation antibacterial strategies against BAIs.

Covalent organic frameworks (COFs) that possess structural stability, post‐modification, outstanding light‐harvesting capacity, and high biocompatibility are promising ROS‐generating antibacterial photosensitizers [10, 11]. A typical design strategy for photosensitive COFs to enhance photocatalytic performance is to incorporate photoactive or electron‐rich units [12], such as porphyrins, phthalocyanines, thiophenes, and triphenylamines [13, 14, 15, 16]. Recently, the use of pillararenes as monomers in highly active COFs has emerged, relying on their sensitive photoelectric and specific molecular recognition properties [17]. Pillararene‐embedded COFs deliver high performance by optimizing both charge dynamics and active‐site accessibility. The incorporated pillararene units facilitate efficient charge‐carrier separation through their rigid and π‐conjugated frameworks. This is coupled with the COF backbone's inherent porosity, which maintains a high density of accessible active sites [18, 19]. Therefore, pillararene‐embedded COFs are envisioned as powerful platforms for photocatalytic antibacterial activity via ROS generation, owing to their pores that facilitate rapid oxygen diffusion and to interactions between ROS and bacteria associated with the pillararene‐embedded COF surface [20, 21].

Photosensitive COF materials can effectively generate ROS for antibacterial purposes. However, the antioxidant defense system within the bacterial infection microenvironment can dampen the bactericidal effect of ROS in multiple ways [22, 23]. For example, enzymatic systems, such as peroxidase, superoxide dismutase, and glutathione peroxidase, can effectively scavenge ROS and reduce their local concentration and bactericidal effect [24]. In addition, small molecule antioxidants (e.g., glutathione and hydrogen sulfide (H_2_S)) can scavenge free radicals to reduce oxidative stress and damage caused by ROS [25, 26, 27]. Thus, an ideal ROS‐based treatment strategy should not only enhance ROS generation but also target antioxidants in the infection microenvironment to further amplify oxidative damage and boost antibacterial efficacy.

The relatively low redox potential of copper ions (Cu^2+^/Cu^+^) (around +0.16 V, much lower than that of iron ions, +0.77 V) means that copper ions can act as mild oxidizing agents in electron transfer reactions (Cu^2+^ + e^−^ → Cu^+^) [28, 29]. This promotes redox activity of copper to modulate redox balance in living systems [30, 31, 32]. Moreover, Cu^2+^ exhibits a strong binding affinity for reducing anionic species, particularly glutathione (GSH) and sulfide ions (S^2−^), enabling selective depletion of antioxidant molecules [33, 34]. Considering the inherent toxicity of free copper ions, encapsulating them within copper‐containing materials, such as copper‐based metal‐organic frameworks (Cu‐MOFs), can be beneficial in biomedicine. Owing to their tunable porosity, high surface area, controlled degradation, and unique biochemical properties, Cu‐MOFs are promising antimicrobial agents that can release copper ions [35]. However, many Cu‐MOFs suffer from low stability due to premature copper leaching and framework collapse, which hampers their antibacterial activity [36]. Thus, the development of copper delivery platforms with robust structures and controlled release profiles is essential to enable the fine‐tuning of copper‐ion delivery, minimize adverse side effects, and ultimately enhance therapeutic efficacy [37]. Thanks to these properties of COFs and MOFs, the combination of the π‐conjugated frameworks of COFs along with the nanoporous structures of Cu‐MOFs could open up a new avenue for the construction of stable and multifunctional multi‐mechanism antibacterial platforms.

**SCHEME 1:**
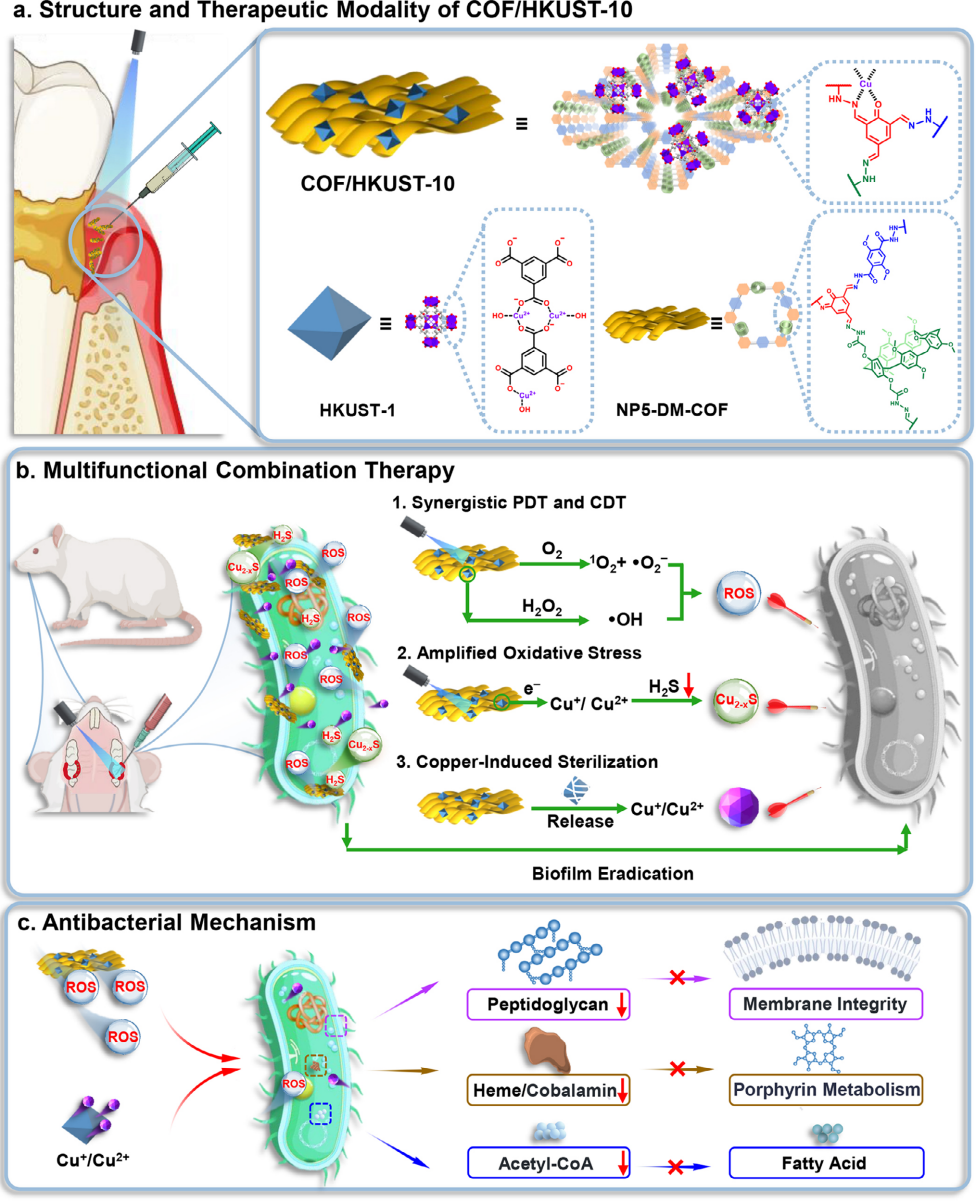
Schematic illustration of the fabrication and anti‐periodontitis application of COF/HKUST‐10. (a) Preparation of COF/HKUST‐10 via in situ deposition of Cu‐MOF onto NP5‐DM‐COF. (b) Schematic representation of the COF/HKUST‐10‐mediated synergistic “three‐in‐one” therapeutic strategy against periodontitis. (c) Antibacterial mechanism of the materials.

Herein, we report a multifunctional supramolecular antibacterial platform designed to deplete H_2_S and amplify oxidative stress for combating periodontitis, a representative BAIs. This platform comprises a COF/HKUST‐10 hybrid in which a Cu‐MOF, HKUST‐1, is in situ generated within a NP5‐DM‐COF (hydrazone‐linked pillararene‐embedded COFs; Scheme 1a). The COF/HKUST‐10 effectively combats BAIs through a “three‐in‐one” antibacterial mechanism (Scheme 1b,c). First, the highly ordered, conductive HKUST‐1 is incorporated into NP5‐DM‐COF to promote ROS generation via synergistic PDT and CDT. Additionally, COF/HKUST‐10 scavenging of H_2_S in the environment weakens its local antioxidant capacity and intensifies oxidative stress. Crucially, ROS‐induced oxidative damage compromises the integrity of bacterial cell membranes, thereby allowing intracellular accumulation of Cu^+^/Cu^2+^, which severely disrupts bacterial metabolism and reduces biofilm colony counts by approximately 4‐log units. This work presents a potential therapeutic strategy for the treatment of BAIs and broadens the prospects of supramolecular materials in biomedical and related fields.

## 2 Results and Discussion

### 2.1 Synthesis, Characterization, and Synergistic Catalysis of COF/HKUST‐10

**FIGURE 1:**
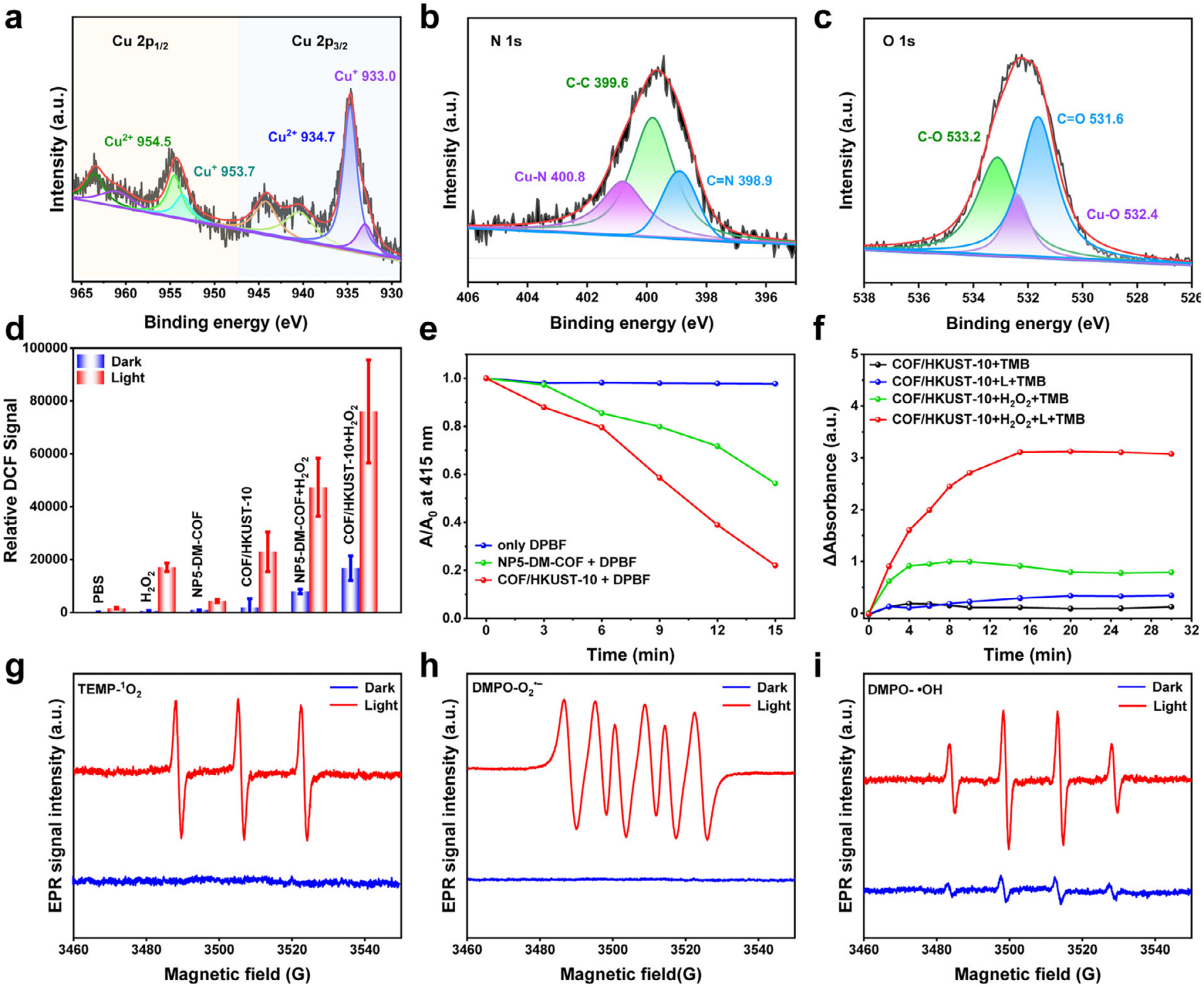
The characterizations of the materials. XPS spectra of (a) Cu 2p, (b) N 1s, and (c) O 1s of COF/HKUST‐10. (d) Total ROS generation under various conditions. (e) Quantification of the ^1^O_2_ generation capabilities of NP5‐DM‐COF and COF/HKUST‐10. (f) Quantification of •OH generation by COF/HKUST‐10 under different conditions using TMB as a probe. (g) EPR spectra of TEMP‐^1^O_2_ under dark and light irradiation. (h) EPR spectra of DMPO−O_2_^•−^ under dark and light irradiation. (i) EPR spectra of DMPO−•OH under dark and light irradiation after incubation of COF/HKUST‐10 with H_2_O_2_.

COF/HKUST‐10 was synthesized using the previously reported two‐step solvothermal method (Scheme S1, Supporting Information) [38, 39]. Structural characterization by powder X‐ray diffraction (PXRD) and Fourier transform infrared (FT‐IR) spectroscopy indicated successful hybridization between NP5‐DM‐COF and HKUST‐1 (Figure S1). To further investigate interfacial interactions and chemical states, X‐ray photoelectron spectroscopy (XPS) was performed. The high‐resolution Cu 2p XPS spectrum of the COF/HKUST‐10 hybrid revealed both Cu^2+^ and Cu^+^ (Figure 1a). These unsaturated Cu^+^ sites likely originate from defective HKUST‐1 material, where the defects are induced by interfacial hybridization. Furthermore, the presence of Cu‐N and Cu‐O bonds has confirmed that hexadentate coordination of Cu^2+^ with carbonyl oxygen and nitrogen atoms in the NP5‐DM‐COF framework (Figure 1b,c). The particle distribution on the surface of NP5‐DM‐COF (Figure S2), along with the clearly visible Cu and O in the elemental mapping of COF/HKUST‐10, also confirmed the successful synthesis of COF/HKUST‐10.

UV–vis diffuse reflectance (UV–vis–DRS) spectroscopy was used to determine the absorption range of COF/HKUST‐10 to be 200–800 nm (Figure S3). Based on the UV–vis absorption analysis of COF/HKUST‐10, low‐power blue LED light irradiation (10 mW cm^−2^, λ = 440 nm) was selected as the optimal excitation source. Experiments were performed to assess the ROS generation ability of NP5‐DM‐COF and COF/HKUST‐10. Initially, the 2′,7′‐dichlorodihydrofluorescein diacetate (DCFH‐DA) assay was employed to quantify total ROS production. As shown in Figure 1d, COF/HKUST‐10 generated significantly higher amounts of ROS compared to NP5‐DM‐COF under light irradiation. This indicates that Cu‐MOF hybridization significantly enhances ROS photogeneration. Notably, when COF/HKUST‐10 was co‐incubated with hydrogen peroxide (H_2_O_2_), ROS production was observed even in the dark, followed by a burst of ROS generation upon light exposure. This phenomenon suggests the COF/HKUST‐10 retains the Cu‐MOF‐mediated Fenton‐like reaction, enabling the hybrid material to exhibit PDT and CDT effects.

We further assessed the ROS generation efficiency of COF/HKUST‐10 using 1,3‐diphenylisobenzofuran (DPBF) and nitroblue tetrazolium (NBT) as selective probes for singlet oxygen (^1^O_2_) and superoxide anions (O_2_^•−^), respectively. As shown in Figure 1e, under 15 min of light irradiation, COF/HKUST‐10 degraded DPBF by 78%, whereas NP5‐DM‐COF degraded DPBF by only 44%. It shows a higher ^1^O_2_‐generation capacity of COF/HKUST‐10 than that of NP5‐DM‐COF. This enhancement can be attributed to the orderedπ‐conjugated structure of COF/HKUST‐10, which facilitates intersystem crossing (ISC) while reducing energy loss during electron transfer [39]. Additionally, COF/HKUST‐10 exhibited photocatalytic degradation of NBT (Figure S4), indicating its ability to generate O_2_^•−^ in aqueous solution under light irradiation.

To elucidate the Fenton‐like activity of COF/HKUST‐10, we examined the classic oxidation of 3,3',5,5'‐tetramethylbenzidine (TMB). COF/HKUST‐10 efficiently catalyzed the decomposition of H_2_O_2_ into the hydroxyl radical (•OH) in a stoichiometric and H_2_O_2_‐concentration‐dependent manner (Figures S5 and S6). Notably, this catalytic activity was significantly enhanced under light irradiation (Figure 1f), highlighting the importance of photo‐enhanced CDT in improving •OH generation. Moreover, the performance of the COF/HKUST‐10 photocatalyst in TMB oxidation and MB degradation experiments is superior to that of NP5‐DM‐COF (Figures S7 and S8). These results suggest that COF/HKUST‐10 generates more ROS and is more photocatalytically active than NP5‐DM‐COF, making it a more effective antibacterial agent in biological systems.

Electron paramagnetic resonance (EPR) spectroscopy identified the specific ROS generated by COF/HKUST‐10. The addition of 2,2,6,6‐tetramethyl‐4‐piperidone (TEMP) and 5,5‐dimethylpyridine‐N‐oxide (DMPO) as trapping agents to the COF/HKUST‐10 suspension resulted in the detection of TEMP‐^1^O_2_ and DMPO‐O_2_^•−^ adducts upon light irradiation (Figure 1g,h). This confirms that COF/HKUST‐10 is a type I and type II photosensitizer, consistent with well‐established photosensitization processes. The DMPO‐•OH adduct signals observed in the presence of H_2_O_2_ were intense both in dark and light (Figure 1i). Therefore, COF/HKUST‐10 is not only an efficient photosensitizer for PDT but also an effective CDT agent that converts H_2_O_2_ to •OH, thereby generating a significant CDT effect. The cooperation between PDT and CDT action greatly enhances ROS generation, thereby improving bacterial efficacy. The NP5‐DM‐COF possesses cavities and functional units that bind copper ions via specific coordination interactions, thus enhancing the coordination stability of HKUST‐1. The presence of HKUST‐1 facilitates charge‐carrier generation, enables charge transfer across interfaces, and prevents the recombination of electrons and holes formed upon light absorption. The interdependence between structural stabilization and functional cooperation between NP5‐DM‐COF and HKUST‐1 maximizes the catalytic performance of PDT and CDT [38, 39].

**FIGURE 2:**
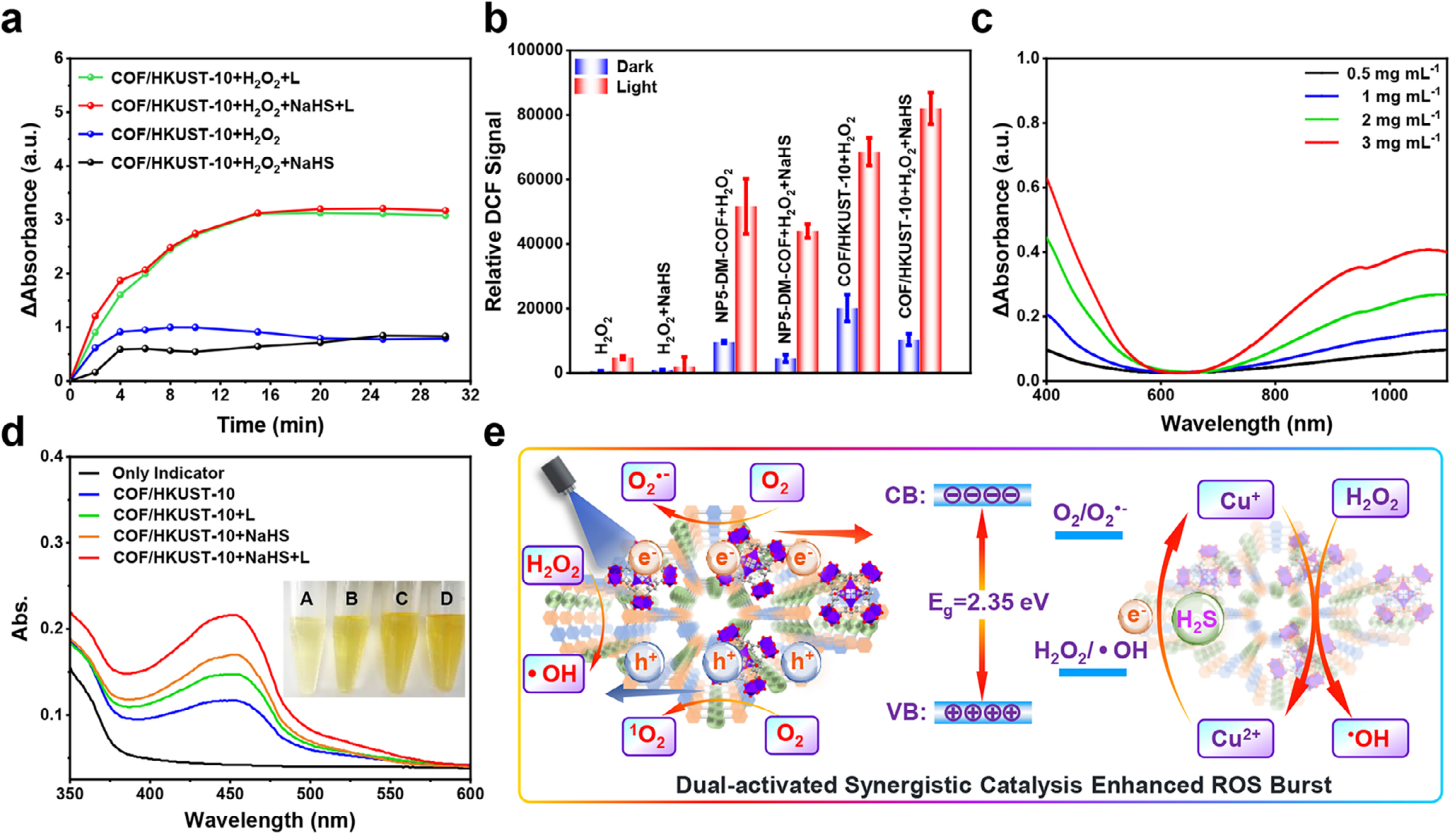
Effect of H_2_S on ROS generation and synergistic catalysis mechanistic investigation. (a) Effect of H_2_S on •OH generation by COF/HKUST‐10 under various conditions. (b) Effect of H_2_S on overall ROS generation. (c) UV–vis–NIR absorbance spectra of COF/HKUST‐10 interacting with H_2_S. (d) UV–vis absorbance spectra of neocuproine after treatment under various conditions. Inset: Photographs of [Cu(neocuproine)_2_]^+^ complex catalyzed by various materials (A: COF/HKUST‐10; B: COF/HKUST‐10 + L; C: COF/HKUST‐10 + NaHS; D: COF/HKUST‐10 + NaHS + L.). (e) Mechanism of dual‐activation synergistic catalysis for amplifying ROS bursts. L: light irradiation.

Despite demonstrating outstanding PDT and CDT efficacy, the influence of microenvironmental antioxidant factors must be considered for practical antibacterial applications of COF/HKUST‐10. H_2_S, a key metabolic product in bacterial infection microenvironments, exhibits stronger antioxidant properties than glutathione, thereby neutralizing ROS and protecting bacteria from oxidative stress [40, 41, 42]. Furthermore, H_2_S can influence bacterial antibiotic resistance and tolerance [43]. It exists as a weak acid in a dynamic equilibrium between H_2_S, the hydrosulfide ion (HS^−^), and the S^2^^−^ ions under physiological conditions [44]. Thus, we simulated endogenous H_2_S under physiological conditions using sodium hydrosulfide (NaHS) and investigated its role in the generation of ROS catalyzed by COF/HKUST‐10. After co‐incubating NP5‐DM‐COF and COF/HKUST‐10 with NaHS (3 mM) for 2 h, TMB probe detection revealed that NaHS did not significantly affect •OH generation by COF/HKUST‐10 under light and dark conditions (Figure 2a), while significantly inhibiting •OH generation by NP5‐DM‐COF (Figure S9).

Interestingly, COF/HKUST‐10 exhibited increased ROS generation under light irradiation (Figure 2b), even though H_2_S is typically an antioxidant and diminishes the PDT and CDT performance of most materials. Total ROS detection indicated that ROS generation was the highest in the presence of NaHS. Subsequently, we investigated the reaction supernatants from the COF/HKUST‐10‐NaHS reaction to elucidate the underlying mechanism. Following the chemical reaction, the absorbance of the supernatant increased significantly in the near‐infrared (NIR) region. This suggests that COF/HKUST‐10 reacts with H_2_S to form Cu_2‐x_S (x denotes the deviation from ideal stoichiometry arising from copper vacancies), which exhibits characteristic NIR absorption due to its localized surface plasmon resonance effect [45, 46]. Moreover, the response of COF/HKUST‐10 to H_2_S displays both concentration‐ and time‐dependent behaviors (Figure 2c; Figure S10). These phenomena can be attributed to several factors. HKUST‐1 decomposes when exposed to water for long enough time to allow Cu^2+^ ions to completely interact with S^2^^−^ ions dissociated from HS^−^ ions. In addition, Cu^2+^ ions prefer the S^2^^−^ ions over the carbonyl O or N atoms of the hydrazone bonds present in the framework. Cu_2‐x_S has a very low solubility product constant. For instance, CuS has a K_sp_ of 6.3 × 10^−36^ [46, 47]. This, together with the above factors, led to the in situ formation of Cu_2‐x_S upon interaction with H_2_S. H_2_S consumption occurs throughout this mechanism, reducing the ability to eliminate ROS and increasing oxidative stress.

To further elucidate the mechanism underlying the dual‐activated synergistic catalysis, we investigated the valence‐state transformation of Cu^+^/Cu^2+^ in COF/HKUST‐10 under light irradiation and H_2_S activation. Neocuproine, a Cu^+^‐specific chelator, was employed as an indicator. Upon binding Cu^+^, colorless neocuproine can form a yellow [Cu(neocuproine)_2_]^+^ complex with a characteristic absorption peak at 452 nm. As shown in Figure 2d, light exposure significantly increased Cu^+^ generation in COF/HKUST‐10. This suggests that photoexcitation of the framework enables the transfer of photogenerated electrons to Cu^2+^, reducing it to catalytically active Cu^+^. For another, although HKUST‐1 is known to decompose under acidic and basic conditions (Figure S11), the structure of COF/HKUST‐10 remained intact after 12 h of immersion in artificial saliva, suggesting that hybridization with NP5‐DM‐COF appears to retard the decomposition rate. This stabilization enables interface‐mediated electron transfer from NP5‐DM‐COF to HKUST‐1 under illumination. This could be attributed to the hydrophobic microenvironment created by the NP5‐DM‐COF framework (water contact angle of 101.6°, Figure S12), which establishes a hydrophobic region rich in benzene rings and methoxy (–OCH_3_) groups around HKUST‐1, thereby retarding the diffusion of H_2_O into the hybrid material. In addition, exposure of COF/HKUST‐10 to NaHS resulted in rapid interaction of HS^−^ with Cu^2+^, leading to Cu^+^ formation. The successful transformation of COF/HKUST‐10 under light and NaHS treatment was confirmed by Cu 2p XPS (Figure S13).

Based on these observations, we propose a mechanism in which COF/HKUST‐10 facilitates photocatalytic ROS generation (Figure 2e). The highly ordered spatial structure and excellent conductivity of HKUST‐1 enhance charge carrier mobility, promote interfacial electron transfer, and suppress the recombination of photogenerated electron‐hole pairs. These characteristics are conducive to ROS generation, which is responsible for PDT effects. Concurrently, within the framework, Cu^2+^ ions can be reduced to Cu^+^ ions by photogenerated electrons and HS^−^ ions. The resulting Cu^+^ species exhibit outstanding catalytic activity, effectively converting H_2_O_2_ into highly toxic •OH radicals, which induce an explosive generation of ROS via CDT effects. Moreover, the consumption of H_2_S during this process reduces its capacity to scavenge ROS, further amplifying oxidative stress. This dual‐activation mechanism provides significant theoretical support for the application of COF/HKUST‐10 composites for antibacterial treatments.

### 2.2 In Vitro Antibacterial Performances of COF/HKUST‐10

H_2_S is a ubiquitous gasotransmitter produced by both anaerobic bacteria (via sulfur‐containing amino acid metabolism) and host cells everywhere in the periodontal microenvironment [48, 49, 50]. Its concentration can notably increase to millimolar levels under pathological conditions, implicating H_2_S in multiple facets of periodontitis progression [51, 52]. Consequently, H_2_S has emerged as a significant biomarker for detection and a promising target for therapeutic intervention [53]. Crucially, H_2_S serves as a universal bacterial defense mechanism against oxidative stress [54, 55]. In this study, we confirmed substantial H_2_S production in major periodontal pathogens using a visual lead acetate detection method. The presence of H_2_S was evidenced by a distinct color change of the test strips from white to black or dark brown (Figure S14). This dual role, as a mediator in periodontitis and an essential bacterial defense, highlights the importance of developing strategies that can effectively modulate H_2_S levels or counteract its effects, especially in conjunction with battling ROS.

**FIGURE 3:**
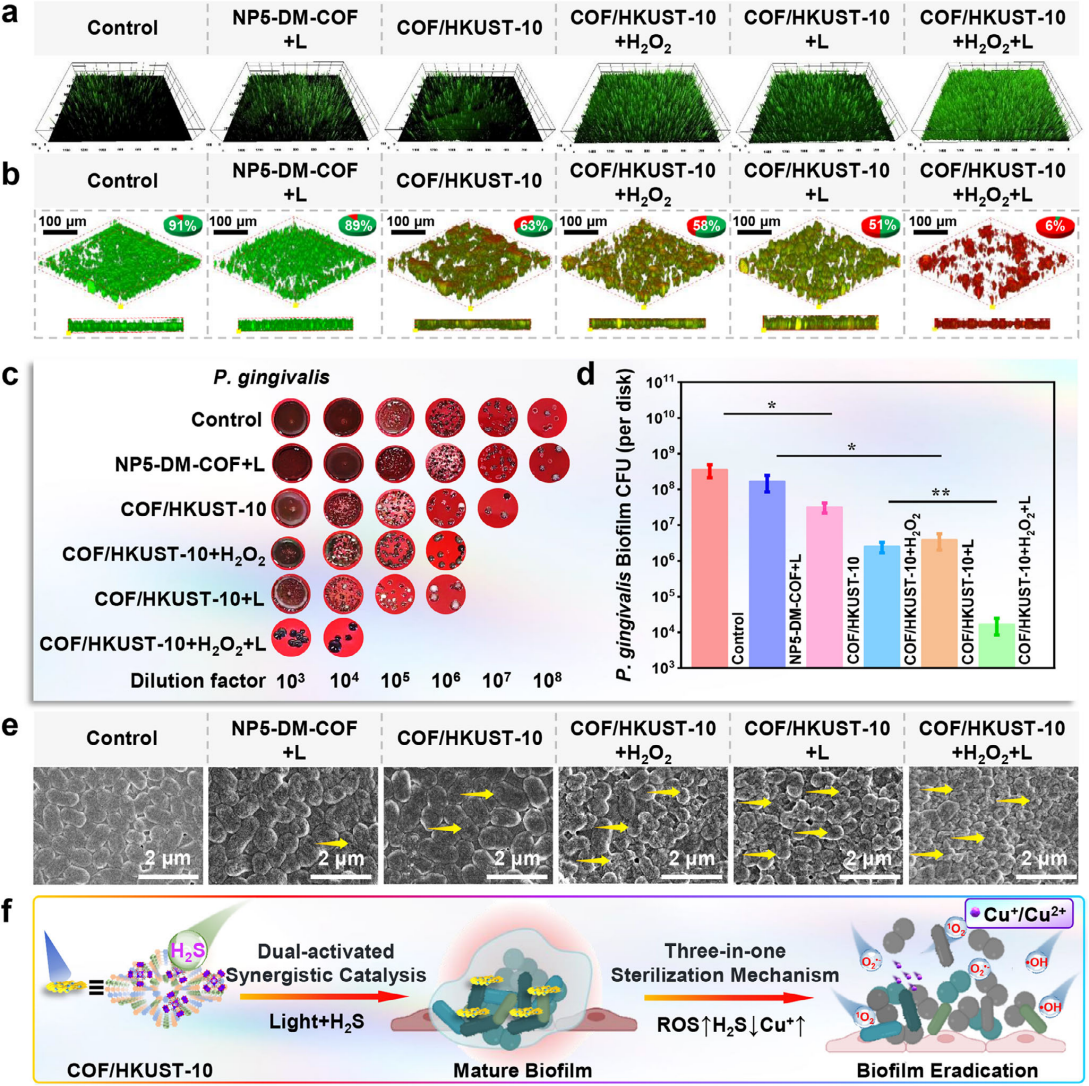
In vitro antibacterial performance of COF/HKUST‐10 under various treatment conditions (H_2_O_2_ concentration: 500 µm where applicable). (a) Fluorescence images of ROS generation in *P. gingivalis* biofilms were detected using the DCFH‐DA probe. (b) 3D CLSM images of live/dead staining of *P. gingivalis* biofilms following various treatments (green: live bacteria, red: dead bacteria). Percentages indicate the proportion of live bacteria within the biofilm. (c, d) Representative images and corresponding quantitative analysis of CFU counts in *P. gingivalis* biofilms under various treatments. (e) SEM images of *P. gingivalis* after various treatments, with yellow arrows indicating the morphology of damaged bacteria. (f) Schematic illustration of the “three‐in‐one” antibacterial mechanism of COF/HKUST‐10 for eradicating plaque biofilms. Data are presented as mean ± SD, *n* ≥ 3; ^*^ *p* < 0.05, ^**^ *p* < 0.01, ^***^ *p* < 0.001; n.s., not significant.

The crucial generation of ROS by COF/HKUST‐10 within biofilms was directly visualized and quantified using the fluorescent probe DCFH‐DA (Figure 3a; Figure S15). We further analyzed the conditional screening of ROS generation. In this case, the composition of COF/HKUST‐10 (structural features) and the inflammatory microenvironment (specific features) are considered. The COF/HKUST‐10 + H_2_O_2_ + L treatment group unequivocally exhibited the strongest fluorescence signal, whereas COF/HKUST‐10 + H_2_O_2_ and COF/HKUST‐10 + L yielded progressively lower signals. In contrast, NP5‐DM‐COF + L and COF/HKUST‐10 alone produced markedly inferior results. Taken together, these data robustly confirm COF/HKUST‐10's capacity for substantial ROS release within biofilms through multimodal synergistic activation under light irradiation.

The antibacterial efficacy of COF/HKUST‐10 was further evaluated for its dispersal of single‐strain biofilm. To assess the biofilm‐disruption effects of treatment, representative 3D live/dead staining was performed (Figure 3b; Figure S16). Despite NP5‐DM‐COF's potential as a photosensitizer for aPDT, the treatment with NP5‐DM‐COF showed limited capacity to eliminate bacterial biofilms at lower concentrations [56]. In the COF/HKUST‐10 treatment group, only a small number of red‐stained (dead) bacteria were observed, indicating modest intrinsic antibacterial activity. However, the subsequent addition of a safe dose of H_2_O_2_ (500 µm) or exposure to light irradiation significantly amplifies the count of red‐stained dead bacteria. Moreover, compared with the control group, the COF/HKUST‐10 + H_2_O_2_ + L group induced significantly higher bacterial cell death and reduced bacterial community density and biofilm thickness (Figure S17). These findings confirm the potent biofilm‐eradicating capability of COF/HKUST‐10, particularly under multi‐mode activation.

In order to more accurately quantify the COF/HKUST‐10's bactericidal effect and biofilm disruption, colony‐forming units (CFUs) assays were undertaken (Figure 3c,d; Figure S18). The biofilm CFU counts were reduced by 1‐log when COF/HKUST‐10 was applied, compared to the control. The reason can be the incorporation of H_2_S into the biofilm, which can reduce the coordinated Cu^2+^ in the COF/HKUST‐10 to toxic Cu^+^. Consequently, this transformation enhances the intrinsic antibacterial properties of the Cu^+^/Cu^2+^ ion [57, 58]. The antibacterial activity of the COF/HKUST‐10 + L group was significantly superior to that of the NP5‐DM‐COF + L group, achieving a 2‐log CFU reduction. This vast improvement indicates that adding Cu‐MOF into the hybrid material enhances the photocatalytic antibacterial activity. A similar 2‐log reduction in CFU count in the presence of H_2_O_2_ further confirms that COF/HKUST‐10 enhances its bactericidal activity through a Fenton‐like reaction mechanism. Crucially, the COF/HKUST‐10 + H_2_O_2_ + L treatment group achieved a 4‐log reduction in CFU counts, demonstrating the most potent bactericidal effect among all conditions evaluated. Bacterial morphology was examined using scanning electron microscopy (SEM) (Figure 3e; Figure S19). In the control group, the bacteria maintained well‐preserved spherical or rod‐like shapes with smooth, intact surfaces. In contrast, various treatment groups exhibited pronounced morphological changes, including cell wall rupture, reduced cellular volume, and structural deformation. Notably, in the COF/HKUST‐10 + H_2_O_2_ + L group, most bacteria showed obvious membrane damage and severely compromised structural integrity.

As a supramolecular antibacterial platform targeting the periodontal microenvironment, COF/HKUST‐10 performs multiple functions in biofilm eradication. Within COF/HKUST‐10, HKUST‐1's highly ordered spatial structure and superior conductivity can effectively inhibit the recombination of photogenerated electron‐hole pairs, significantly enhancing charge carrier transport efficiency. Such characteristics imply that COF/HKUST‐10 can circumvent the limitations of low oxygen tension, as its intrinsic charge‐carrier migration capabilities may facilitate the generation of reactive species through more oxygen‐independent mechanisms. Second, COF/HKUST‐10 takes advantage of HKUST‐1's classic Fenton‐like property. The Cu^2+^ ions in the structure are effectively reduced to Cu^+^ by photogenerated charge carriers and H_2_S, enabling the synergistic catalysis of PDT and CDT. Furthermore, COF/HKUST‐10 can effectively detoxify H_2_S due to the comparatively weak Cu─O coordination bonds in its MOF framework and the extremely low solubility product constant of Cu^2+^ with S^2−^ ions. This action reduces the antioxidant capacity, thereby further amplifying oxidative stress. Moreover, this synergistic catalytic mechanism allows COF/HKUST‐10 to disrupt biofilm integrity and oxidize bacterial cell membrane lipids and proteins, consequently increasing membrane permeability. Damage to this membrane promotes the entry of Cu^+^/Cu^2+^ ions into bacterial cells. Integrating the synergistic catalytic effects of PDT and CDT with the inherent antibacterial properties of Cu^+^/Cu^2+^ ions, COF/HKUST‐10 serves as an ideal instance of “three‐in‐one” synergistic mechanism for effective bacterial death and biofilm eradication (Figure 3f).

**FIGURE 4:**
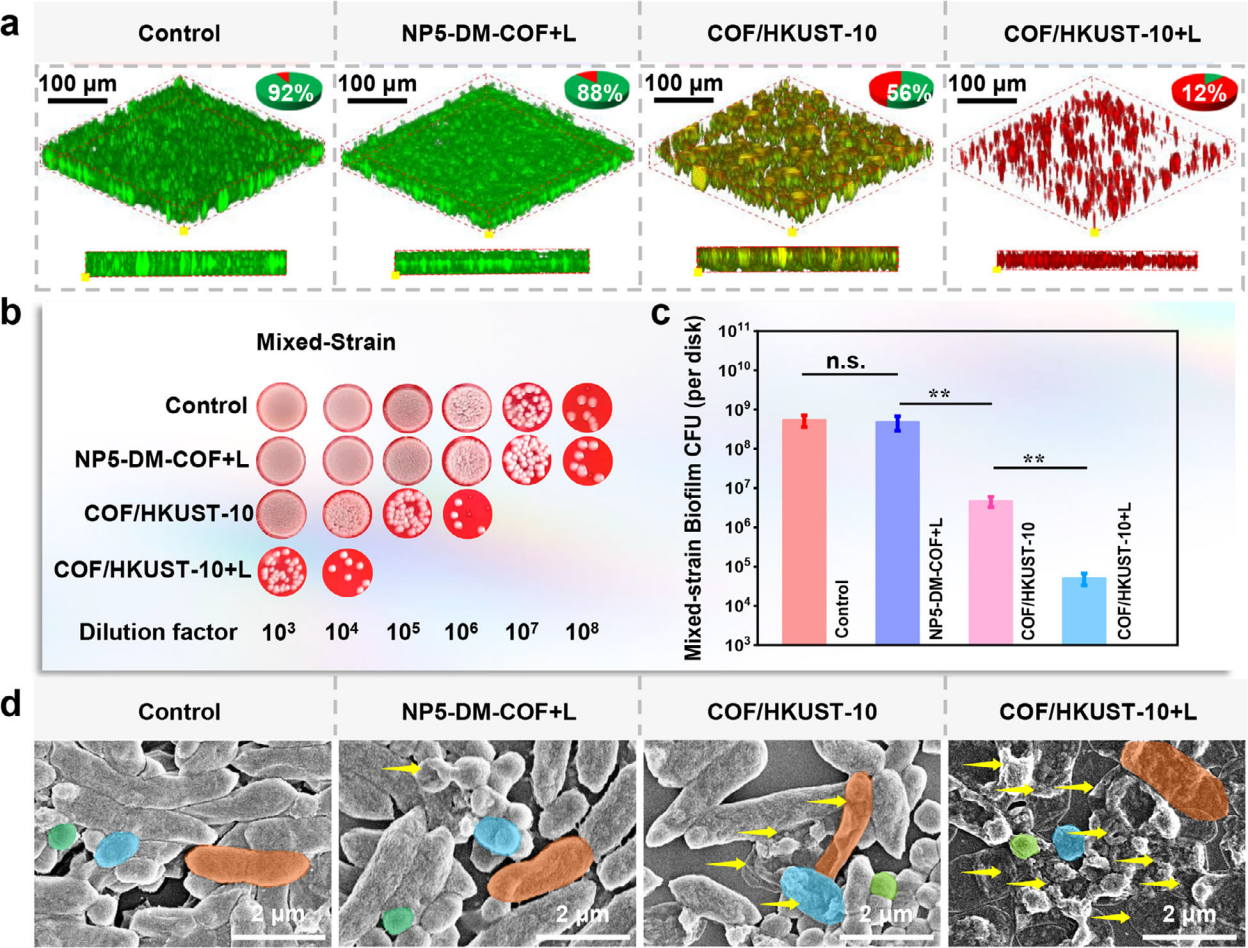
In vitro antibacterial performance of COF/HKUST‐10 against mixed‐strain biofilms. (a) 3D CLSM images of live/dead staining of mixed‐strain biofilms following various treatments (green: live bacteria, red: dead bacteria). Percentages indicate the proportion of live bacteria within the biofilm. (b, c) Representative images and corresponding quantitative analysis of CFU counts in mixed‐strain biofilms under various treatments. (d) SEM images of mixed‐strain under various treatments. Green: *S. gordonii*, blue: *P. gingivalis*, orange: *F. nucleatum*. Data are presented as mean ± SD, *n* ≥ 3; ^*^ *p* < 0.05, ^**^ *p* < 0.01, ^***^ *p* < 0.001; n.s., not significant.

Bacterial aggregation in biofilms forms complex 3D structures, significantly complicating BAIs [59, 60]. For multilayered periodontal bacterial biofilms, *Streptococcus gordonii* (*S. gordonii*) is an early colonizer that stabilizes the biofilm by secreting adhesion factors and extracellular matrix components [61]. *S. gordonii* is also a prominent H_2_O_2_ producer with diverse activity in periodontitis initiation and progression [62]. We established a complex oral biofilm model using *S. gordonii*, *P. gingivalis*, and *F. nucleatum* to evaluate the efficacy of COF/HKUST‐10 in eradicating these biofilms. The findings from the 3D live/dead staining indicated that biofilm architecture was damaged for both the COF/HKUST‐10 and COF/HKUST‐10 + L groups, with a significant decrease in viable bacteria and a distinct decline in biofilm thickness (Figure 4a; Figure S20). CFU quantification confirmed that the COF/HKUST‐10 + L group demonstrated a stronger antibacterial effect than the COF/HKUST‐10 group (Figure 4b,c). In the COF/HKUST‐10 + L group, SEM images showed that bacteria sustained severe damage to the membrane and structural disintegration (Figure 4d). These results indicate that COF/HKUST‐10 has strong bactericidal and biofilm‐disrupting ability in a complex mixed‐strain biofilm.

### 2.3 Antibacterial Mechanism of COF/HKUST‐10

**FIGURE 5:**
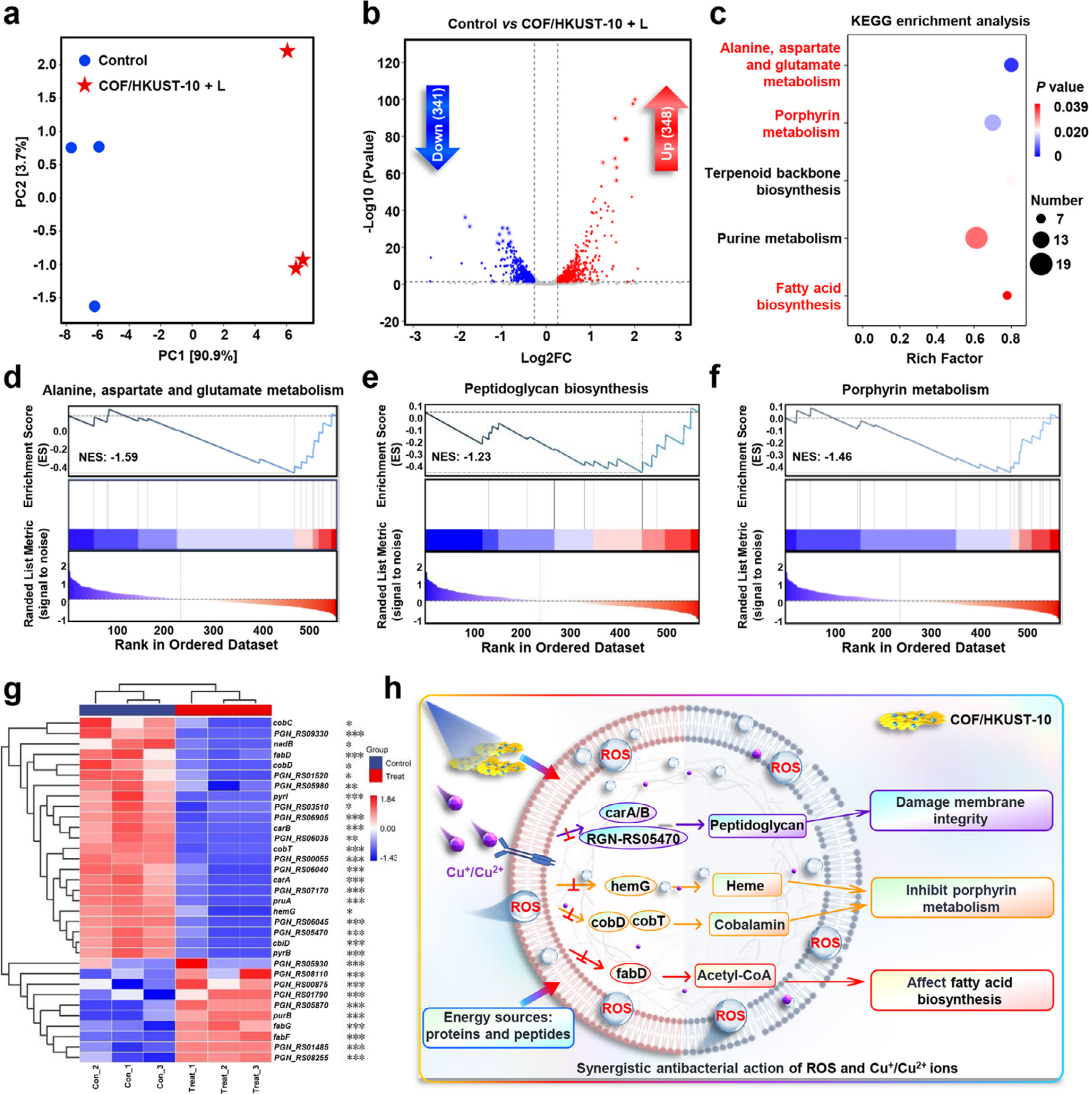
Transcriptomic analysis of *P. gingivalis* following treatment. (a) PCA of transcriptomic profiles. (b) Volcano plot showing the distribution of DEGs between the COF/HKUST‐10 + L and control groups. (c) KEGG enrichment analysis comparing the control and COF/HKUST‐10 + L treatments. (d) Gene set enrichment analysis (GSEA) of the alanine, aspartate, and glutamate metabolism pathway. (e) GSEA of the peptidoglycan metabolism pathway. (f) GSEA of the porphyrin metabolism pathway. (g) Heatmap of major DEGs showing expression patterns under different treatments. (h) Schematic illustration of the proposed antibacterial mechanism of COF/HKUST‐10. Data are presented as mean ± SD, *n* ≥ 3, ^*^ *p* < 0.05, ^**^ *p* < 0.01, ^***^ *p* < 0.001.

To elucidate the antibacterial mechanism of COF/HKUST‐10, transcriptomic analysis of *P. gingivalis* treated with COF/HKUST‐10 + L was carried out based on an untreated control group. The principal component analysis (PCA) between biological replicates of each group was highly reproducible with no outliers (Figure 5a). After analyzing volcano plots, a total of 689 differentially expressed genes (DEGs) were identified in the COF/HKUST‐10 + L, which is 348 upregulated and 341 downregulated as compared to the control (Figure 5b). Gene ontology (GO) enrichment analysis revealed that COF/HKUST‐10 + L treatment significantly modulated diverse biological functions in *P. gingivalis*, including aromatic compound biosynthesis, ribosomal subunit assembly, DNA‐templated transcription, and signal transduction (Figure S21). These suggest a substantial disruption of *P. gingivalis'* cellular metabolism, physiological activities, and gene regulatory processes associated with its pathogenicity. Kyoto encyclopedia of genes and genomes (KEGG) enrichment analysis elucidated significant biological networks, including metabolic pathways, signal transduction pathways, and cellular processes. These pathway maps are essential for unveiling cellular and molecular mechanisms and establishing examination connections among metabolic pathways [63]. The corresponding KEGG plot revealed that COF/HKUST‐10 + L treatment markedly influenced several metabolic pathways, including alanine, aspartate, and glutamate metabolism; porphyrin and purine metabolism; and fatty acid biosynthesis (Figure 5c).

Unlike many other bacteria, *P. gingivalis* is a strictly anaerobic organism that requires an oxygen‐free environment for metabolism. It lacks a classical tricarboxylic acid cycle or electron transfer chain. Instead, it primarily derives energy from proteins and peptides. Analysis of the amino acid and peptide metabolism of the pathogen is critical for sustaining energy requirements and virulence expression [64, 65, 66]. Alanine and peptidoglycan are crucial structural components of the gram‐negative bacterial cell wall. It is essential for maintaining cell wall integrity [67]. COF/HKUST‐10 + L is proven to inhibit alanine and peptidoglycan biosynthesis, leading to disruption of cell wall synthesis and structural integrity, which in turn increases membrane permeability (Figure 5d,e). This inhibition of metabolic pathways may be associated with ROS interference [68]. Notably, in porphyromonas gingivalis treated with COF/HKUST‐10, the inhibition of peptidoglycan synthesis halts structural remodeling rather than mediating direct enzymatic degradation of the established layer. While extensive peptidoglycan loss is not observed, this inhibition significantly sensitizes the bacteria to osmotic lysis. The resulting compromise in the layer's mechanical resilience, in concert with enhanced outer membrane permeability, ultimately culminates in a catastrophic collapse of cell envelope integrity. The simultaneous inhibition of aspartate and glutamate biosynthesis depletes the energy supply of the bacterium, diminishes its antioxidant ability, and halts the expression of virulence factors.

*P. gingivalis* is highly dependent on iron, particularly in the form of heme (iron protoporphyrin), so porphyrin metabolism is essential for its viability and virulence [69, 70]. The hem gene family is the primary locus in this biosynthetic pathway, and a multistep enzymatic conversion of glutamate to heme occurs. Heme‐associated enzymes often contain cysteine residues that are essential for catalysis. Their thiol groups (─SH) are highly susceptible to oxidation into disulfide bonds (─S─S─) or coordination with metal ions [71, 72]. Such modifications can induce conformational changes or affect the active‐site structure, ultimately attenuating its catalytic efficiency. Additionally, cobalamin (vitamin B_12_), a cobalt‐containing coenzyme, is proposed to be a critical regulator in *P. gingivalis*, potentially modulating porphyrin metabolism and associated enzymatic processes [73]. Cobalamin biosynthesis genes and the hem gene family are significantly downregulated upon COF/HKUST‐10 and light irradiation by transcriptomic analysis (Figure S22). Furthermore, pathway enrichment analysis confirmed a significant suppression of the porphyrin metabolic pathway (Figure 5f). It is said that this inhibition may occur through the combination of ROS and Cu^+^/Cu^2+^ ions, which can oxidize or coordinate important cysteine residues. Interactions of these chemicals disrupt the activity of essential enzymes involved in heme and cobalamin biosynthesis [74, 75, 76]. Such enzymatic inhibition blocks the porphyrin and heme biosynthetic pathways and substantially inhibits bacterial growth [77]. In addition, inhibition of fatty acid biosynthesis and blockade of purine metabolism further choke their growth and pathogenicity (Figure S23). Moreover, the downregulation of the two‐component system can significantly affect energy metabolism and environmental sensing of bacteria (Figure S24). The gene expression profiles of the COF/HKUST‐10 + L‐treated group and the control group show enormous differences as per heatmap analysis (Figure 5g).

Transcriptomic investigation reveals that the antibacterial action of COF/HKUST‐10 against *P. gingivalis* is primarily due to the synergistic effect of ROS and Cu^+^/Cu^2+^ ions (Figure 5h). As a result, cell membrane integrity, porphyrin metabolism, fatty acid biosynthesis, and essential amino acids synthesis are affected. The metabolic disruptions can significantly impede bacterial growth, reduce environmental adaptability, diminish pathogenicity to the host, and impair biofilm formation. Therefore, the ability of *P. gingivalis* to survive and colonize the oral cavity is significantly diminished, which, in turn, reduces its pathogenicity in periodontal disease.

### 2.4 In Vivo Antibacterial Performances of COF/HKUST‐10

**FIGURE 6:**
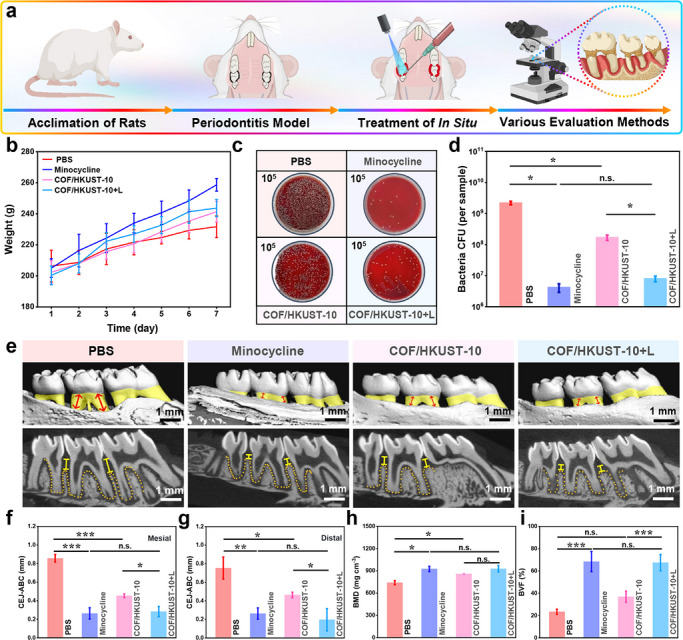
In vivo antibacterial performances of COF/HKUST‐10 in a ligature‐induced periodontitis rat model. (a) Schematic diagram depicting the establishment, administration, and treatment of the ligature‐induced periodontitis model. (b) Changes in body weight of rats across different treatments. (c) Representative images of bacterial colonies isolated from the gingival tissues. The number in the upper left corner represents the dilution factor. (d) Quantitative analysis of CFU counts. (e) Micro‐CT and 3D reconstructed images showing alveolar bone loss. Red arrows indicate the CEJ‐ABC distance. (f) Quantitative measurement of CEJ‐ABC at the mesial site. (g) Quantitative measurements of CEJ‐ABC at the distal site. (h) BMD following treatment. (i) BVF following treatment. Data are presented as mean ± SD, *n* ≥ 3; ^*^ *p* < 0.05, ^**^ *p* < 0.01, ^***^ *p* < 0.001; n.s., not significant.

Prompted by the potent in vitro antibacterial activity of COF/HKUST‐10, we further evaluated its in vivo antibacterial efficacy and anti‐inflammatory effects using a rat periodontitis model (Figure 6a). This model was established through ligation combined with oral inoculation of a mixed bacterial suspension. The experimental protocol was approved by the Institutional Animal Care and Use Committee (IACUC) of the School of Basic Medical Sciences, Jilin University (Approval No. 2025–436, Changchun, China). Periodontitis‐bearing rats were randomly assigned to four treatment groups: PBS, minocycline hydrochloride, COF/HKUST‐10, and COF/HKUST‐10 + L. Treatments were administered three times on an every‐other‐day schedule. Animal body weight was monitored throughout the experiment. To ensure objectivity, a double‐blind methodology was implemented for both Micro‐CT and histological analyses.

Rats across all groups maintained good general health, evidenced by normal appetite and consistent body weight gain throughout the experiment (Figure 6b). This suggests favorable local biocompatibility of COF/HKUST‐10 and minimal systemic physiological impact. To assess the in vivo antibacterial efficacy of COF/HKUST‐10, subgingival bacteria were collected post‐treatment and cultured on Columbia blood agar plates (Figure 6c,d). According to in vitro results, the COF/HKUST‐10 + L group exhibited a significant decrease in pathogenic bacterial load as compared to the untreated periodontitis control. Significantly, the material exhibits antibacterial efficacy comparable to minocycline, suggesting therapeutic potential. The potential therapeutic value of COF/HKUST‐10 is further indicated by the known biological role of copper in the regulation of innate immunity [30]. Infection of phagocytic cells, such as macrophages, stimulates the production of copper transporters (Ctr1 and ATP7A) and copper chaperones (Atox1) [78]. Copper ions are then actively taken up through these transporters. ATP7A is a copper transporter that resides in the Golgi apparatus and is trafficked to phagosomes, where it causes the release of copper ions into the lumen of pathogen‐containing vesicles, thus boosting local antimicrobial activity. Research has shown that high levels of Cu^2+^ in saliva are associated with less decay [79]. This could have clinical implications for the ability of copper ions to hinder the growth of mouth bacteria.

The distance from the cementoenamel junction to the alveolar bone crest (CEJ‐ABC) is an important parameter for assessing changes in alveolar bone level. A higher CEJ‐ABC distance indicates greater bone loss due to periodontitis. Different treatment groups can be compared using CEJ‐ABC measurements, which enable a quantitative assessment of bone destruction and disease severity. Micro‐CT imaging showed that the CEJ‐ABC distance at the mesial aspect of the second molar was increased in the PBS group, as were the values at the distal aspect (indicating severe alveolar bone loss) (Figure 6e–g). Conversely, the CEJ‐ABC distance in COF/HKUST‐10 + L was significantly shorter due to less alveolar bone resorption. The findings confirm the potential of COF/HKUST‐10 + L to treat periodontal inflammation and inflammatory bone resorptions. Moreover, this group showed enhanced bone preservation or suppression of resorptive activity, as evidenced by the significant upsurge in bone mineral density (BMD) and bone volume fraction (BVF) (Figure 6h,i). The above results further indicate that COF/HKUST‐10 prevents alveolar bone loss through local anti‐inflammation.

**FIGURE 7:**
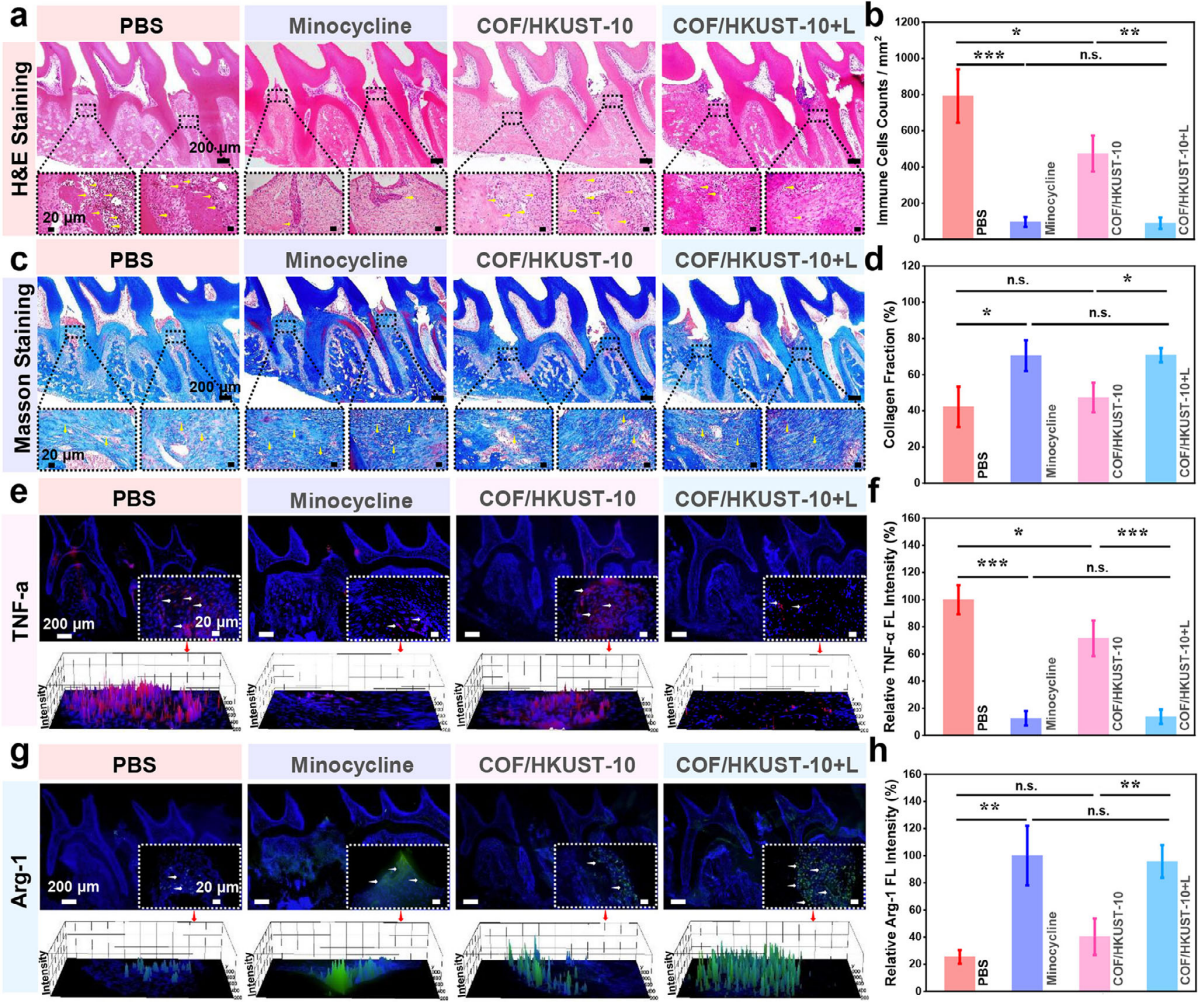
Anti‐infective performance in ligature‐induced periodontitis model. (a) H&E staining showing infiltration of inflammatory cells. Scale bars are 200 (full view) and 20 (enlarged view) nm. (b) Quantitative analysis of immune cell infiltration. (c) Representative images of Masson's trichrome staining. Scale bars are 200 (full view) and 20 (enlarged view) nm. (d) Quantitative evaluation of collagen content. (e) Immunofluorescence images showing TNF‐α expression. Scale bars are 200 (full view) and 20 (enlarged view) nm. (f) Quantitative analysis of TNF‐α immunofluorescence intensity. (g) Immunofluorescence images showing Arg‐1 expression. Scale bars are 200 (full view) and 20 (enlarged view) nm. (h) Quantitative analysis of Arg‐1 immunofluorescence intensity. Yellow arrows indicate representative staining results. Data are presented as mean ± SD, *n* ≥ 3, ^*^ *p* < 0.05, ^**^ *p* < 0.01, ^***^ *p* < 0.001, n.s., not significant.

We performed hematoxylin–eosin (H&E) and Masson's trichrome staining for a comprehensive evaluation of the inflammatory status. The PBS group showed extensive infiltration of inflammatory cells (yellow arrows, Figure 7a,b), indicating a high incidence of inflammation and severe tissue damage. By contrast, the treatment group COF/HKUST‐10 + L exhibited fewer inflammatory cells, indicating effective inhibition of local inflammation. Masson's trichrome staining exhibited the collagen fibers and other tissue components, providing insight into tissue remodeling. The PBS group contains a loose connective tissue structure along with an unclear arrangement of collagen fibers, signifying acute damage and cellular infiltration. The absence of structural disarray is primarily due to cytokines such as IL‐1 and TNF‐α. Cytokines, produced by neutrophils as well as macrophages that infiltrate tissue, inhibit collagen synthesis and stimulate its degradation. In contrast, the arrangement of collagen fibers was dense, well‐organized, and interlaced for the COF/HKUST‐10 + L and antibiotic‐treated groups (highlighted by yellow arrows, Figure 7c,d), indicating tissue repair.

To further elucidate the anti‐inflammatory mechanism by conducting immunofluorescence staining of TNF‐α (pro‐inflammatory) and Arg‐1 (anti‐inflammatory). PBS group exhibited an increase in TNF‐α expression, suggesting a sustained inflammatory response and ongoing tissue injury (yellow arrows, Figure 7e,f). On the other hand, the Arg‐1 expression of COF/HKUST‐10 + L and the antibiotic group is increased (marked with yellow arrows in Figure 7g,h). The pro‐inflammatory substances can be inhibited, and overactivation of the immune response can be reduced. Furthermore, this may also help in tissue damage as well. Research shows that COF/HKUST‐10, when exposed to the appropriate light stimulus, exhibits strong antibacterial activity against periodontal pathogens and a strong anti‐inflammatory response. This two‐pronged action takes advantage of the inherent property of copper and, together with the host immune reaction, offers a therapeutic option for inflammation and healing in oral infections

To assess the potential cumulative toxicity of COF/HKUST‐10 in vivo, H&E staining was performed on the main organs. The absence of noticeable histopathological changes and inflammatory responses, as shown in Figure S25, demonstrates that COF/HKUST‐10 possesses good biocompatibility and systemic safety. The cytocompatibility of COF/HKUST‐10 was further examined against L929 cells using the CCK‐8 assay (Figure S26). Exposure to COF/HKUST‐10 at concentrations up to 50 µg mL^−1^ resulted in cell viability of more than 90% after 24 h of continuous exposure with or without light. To assess blood compatibility, hemolysis assays were conducted. At a 100 µg mL^−1^ sample concentration, the hemolysis rate was less than 3% (Figure S27), which is within acceptable limits for biomedical applications. Overall, these results confirm that COF/HKUST‐10 is an excellent biocompatible and low systemic toxic material for the treatment of periodontal disease.

## 3 Conclusion

In summary, we have successfully developed COF/HKUST‐10, a dual‐activated antibacterial platform tailor‐made to target H_2_S in the periodontitis microenvironment and eliminate BAIs. The rational design and facile synthesis of this supramolecular architecture combine the structural tunability and synergistic benefits of Cu‐MOF and NP5‐DM‐COF. Healing defects and functional complementarity significantly advance the photocatalytic performance and thus produce composite effects to boost the “three‐in‐one” antibacterial mechanism rather than only additive ones: (i) In dual photoactivation and the elevated H_2_S concentration in the periodontitis microenvironment, COF/HKUST‐10 renders synergistic PDT and CDT for efficient ROS generation. (ii) COF/HKUST‐10 scavenges H_2_S, thereby mitigating its ROS‐scavenging capabilities and further augmenting local oxidative stress. (iii) Generated ROS causes disruption of the bacterial cell wall, membrane permeability, and uptake of Cu^+^/Cu^2+^ ions inside the cell. This, in turn, disrupts *P. gingivalis* metabolism, leading to bacterial death. The COF/HKUST‐10 exhibits potent in vitro biofilm eradication ability owing to the synergistic effects of ROS and Cu^+^/Cu^2+^ ions. In vivo experiments further confirm its good biocompatibility and safety, showing significant reduction of pathogenic bacteria loading, alleviation of inflammation, and promotion of collagen deposition, thus providing an effective strategy against periodontal infection. This study presents an efficient method for treating periodontitis and other BAIs and broadens the application of supramolecular materials.

## Author Contributions

S.L., M.‐H.L., L.W., and Y.‐W.Y. conceived and designed the experiments. S.L., M.‐H.L., Y.W., and H.H. conducted the material characterizations. S.L., L.C., T.‐S.Z., H.‐P.Z., and B.‐W.L. performed the biological experiments. S.L. and M.‐H.L. analyzed the experimental data and co‐wrote the manuscript. L.W. and Y.‐W.Y. supervised the project and revised the manuscript. All authors have approved the final version of the manuscript. S.L. and M.‐H.L. contributed equally.

## Conflicts of Interest

The authors declare no conflict of interest.

## Supporting information




**Supporting File**: advs75608‐sup‐0001‐SuppMat.docx.

## Data Availability

The data that support the findings of this study are available from the corresponding author upon reasonable request.
