# Supplementary material for: H2S‐Amplified “Three‐in‐One” Antibacterial Strategy for Periodontitis Treatment Using a Photosensitive Pillararene‐Embedded COF/MOF Hybrid
Source: Adv Sci (Weinh). 2026 May 7;13(43):e75608. doi: 10.1002/advs.75608 (PMC13335815; doi:10.1002/advs.75608)
Supplement: Supplementary file 1 — Supporting File: advs75608‐sup‐0001‐SuppMat.docx. [file ADVS-13-e75608-s001.docx]

Supporting Information

H_2_S-Amplified “Three-in-One” Antibacterial Strategy for Periodontitis Treatment Using a Photosensitive Pillararene-Embedded COF/MOF Hybrid

*Shuang Liang, Meng-Hao Li, Liang Cheng, Tian-Shou Zhang, Hui Hui, Yan Wang, Hong-Pu Zhang,* *Bo-Wen Liu, Lin Wang,* and Ying-Wei Yang**

S. Liang, L. Cheng, T.-S. Zhang, H.-P. Zhang, B.-W. Liu, Prof. L.-Wang

Department of Prosthodontics, Jilin Provincial Key Laboratory of Sciences and Technology for Stomatology Nanoengineering, School and Hospital of Stomatology, Jilin University, Changchun 130021, P. R. China

E-mail: wanglin1982@jlu.edu.cn (L.W.)

S. Liang, Dr. M.-H. Li, H. Hui, Prof. Y. Wang, Prof. Y.-W. Yang

College of Chemistry, Jilin University, 2699 Qianjin Street, Changchun 130012, P. R. China

E-mail: ywyang@jlu.edu.cn (Y.-W.Y.)

**Table of Contents**

Section 1. Materials and instruments….…………….…………….…...………...………...S3

Section 2. Synthesis procedures ………………………...………..…..…….…….………...S5

Section 3. Photocatalytic performance and H_2_S-responsiveness of COF/HKUST-10...…..S6

Section 4. *In vitro* antibacterial experiment………………..................................................S7

Section 5. Transcriptomic analysis……………………..…………...…..….……….............S9

Section 6 *In vivo* antibacterial experiment.…………….………………….……….............S10

Section 7. Biosafety assay of COF/HKUST-10...................................…...………................S11

Section 8. Statistical analysis……………………………………………………………....S12

Section 9. Supporting figures………………………………………...……….…………...S12

References………………………………………………………………...………………...S22

**Section 1. Materials and instruments**

**1.1 Materials and reagents**

Copper acetate monohydrate, trimesic acid, and glacial acetic acid were purchased from Aladdin (Shanghai, China). Neocuproine, methylene blue (MB), sodium hydrosulfide (NaHS), 1,3-Diphenylisobenzo-furan (DPBF), N-Methyl-2-pyrrolidone (NMP), Nitro blue tetrazolium (NBT), and 2′,7′-dichlorofluorescein diacetate (DCFH-DA) were purchased from Aladdin (Shanghai, China). 3,3',5,5'-Tetramethylbenzidine (TMB) was purchased from Aladdin Reagent Co., Ltd (Shanghai, China). 5,5-Dimethyl-l-pyrroline-N-oxide (DMPO) and 2,2,6,6-tetramethylpiperidine (TEMP) were purchased from Sigma (St. Louis, MO, USA). 4% Paraformaldehyde fix solution (PFA) and Triton-X-100 were purchased from Beyotime (Beijing, China). Arginase 1 antibody: sc-271430 and anti-TNF-α (52b83): sc-52746 were purchased from Santa Cruz Biotechnology, Inc (Shanghai, China). Dulbecco’s Modified Eagle Medium (DMEM) and fetal bovine serum (FBS) were obtained from Gibco (Grand Island, NY, USA). Penicillin-Streptomycin Solution were purchased from Hyclone (GE Healthcare, Logan, UT, USA). Tryptic soy broth (TSB), menadione, yeast extract, L-cysteine hydro­chloride, hemin, and Tris-HCl were purchased from Sigma-Aldrich (St. Louis, MO, USA). Columbia blood agar was purchased from BIO-KONT (Wenzhou, China). Cell Counting Kit-8 (CCK-8) was obtained from Beijing Solarbio. 4′,6-Diamidino-2-phenylindole (DAPI) and SYTOX were purchased from Sigma-Aldrich (St. Louis, MO, USA). All bacteria strains were obtained from American Type Culture Collection (ATCC, Manassas, VA): *Porphyromonas gingivalis* (*P. gingivalis*) ATCC 33277 and *Fusobacterium nucleatum* (*F. nucleatum*) ATCC 25586, *Streptococcus gordonii* (*S. gordonii*) ATCC10558. All other biological kits were obtained from Sigma-Aldrich (St. Louis, MO, USA) except as otherwise noted.

**1.2 Instruments and methods**

**1.2.1 Fourier transform infrared spectroscopy (FT-IR)**

FT-IR spectra were tested with a Thermo Fisher Nicolet iS5 spectrometer. The samples were mixed with KBr powder to make transparent sheets for testing in the range of 500–4000 cm^-1^.

**1.2.2 Scanning electron microscopy (SEM)**

SEM images were carried out using a Hitachi SU8020 microscope.

**1.2.3 Powder X-ray diffraction (PXRD)**

PXRD patterns were carried out on a PANalytical B.V. Empyrean diffractometer.

**1.2.4 UV-visible (UV-vis) absorption spectra (Liquid)**

UV-vis absorption spectra (liquid) were recorded on a JASCO model V-670 spectrometer.

**1.2.5 UV-visible (UV-vis) absorption spectra (Solid)**

UV-visible absorption spectra (solid) of the materials were carried out on a Perkin Elmer Lambda 950 spectrometer by measuring the diffuse reflectance of the powders.

**1.2.6 X-ray photoelectron spectroscopy (XPS)**

The X-ray photoelectron spectroscopy was measured on a Thermo Scientific K-Alpha by sticking the powder sample onto a conductive paste.

**1.2.7 Element analysis test**

Energy dispersive spectroscopy (EDS) images of C, N, O, and Cu were recorded on a HITACHI SU8082 instrument.

**1.2.8 Electron paramagnetic resonance (EPR) test**

The EPR spectra were acquired using a Bruker ELEXSYS E500 EPR spectrometer. The EPR-spin trapping technique coupled with the spin traps DMPO and TEMP was used to detect hydroxyl radicals (•OH), superoxide radical (O_2_^•−^), and singlet oxygen (^1^O_2_). The EPR signals were recorded under both dark and light irradiation conditions.

**1.2.9 Confocal laser scanning fluorescence microscope (CLSM)**

The fluorescence measurements were conducted by a confocal laser scanning fluorescence microscope (CLSM, FV1200, Olympus, Japan).

**1.2.10 Microplate reader**

The microplate reader is a UV/Vis microplate spectrophotometer capable of detecting all photometric measurements (SpectraMax M5, Molecular Devices, Sunnyvale, CA, USA).

**1.2.11 Micro-computed tomography (Micro-CT) scanner**

The hard tissues containing maxillary and teeth were scanned with a Micro-CT scanner (Scanco Medical AG, Bassersdorf, Switzerland) to assess bone loss. The vertical distance between the alveolar bone crest (ABC) and the cementoenamel junction (CEJ) in the maxillary second molar was calculated as a measure of bone loss following 3D CT image reconstruction.

**1.2.12 Histological evaluation**

Hematoxylin and eosin (H&E) staining, Masson's trichrome staining, and immunofluorescent staining were used on the scanned maxillary tissues to analyze inflammation and tissue repair, respectively.

**Section 2. Synthesis of COF/HKUST-10**

**
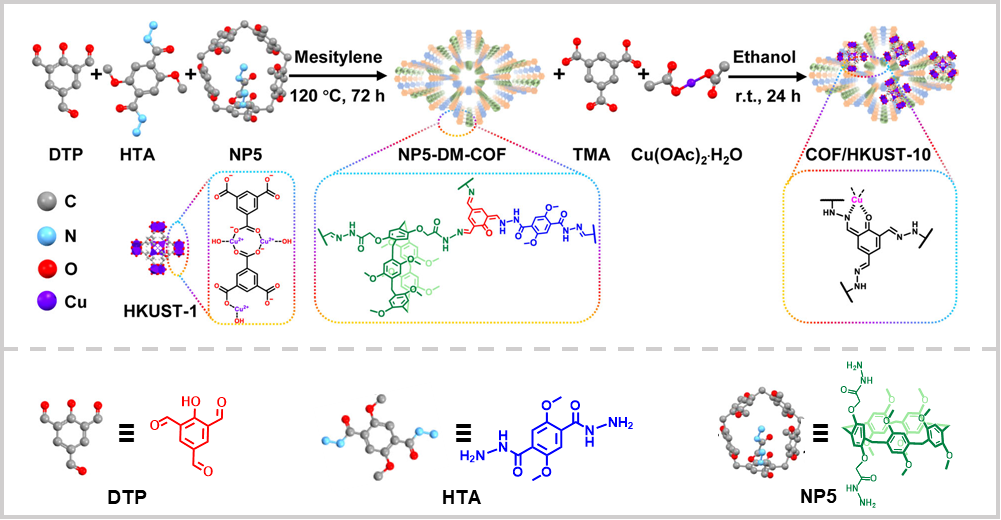
**

**Scheme S1.** Synthetic route of COF/HKUST-10.

NP5-DM-COF was synthesized according to our published procedure.^[S1,S2]^ Briefly, NP5-DM-COF was synthesized from 2-hydroxy-1,3,5-tricarbaldehyde (HTA), 2,5-dimethoxyterephthalohydrazide (DTP), and hydrazine-functionalized pillararene (NP5) in a molar ratio of 4:3:3 (HTA/DTP/NP5) by solvothermal condensation.

COF/HKUST-10 was synthesized according to our published procedure.^[S3]^ Firstly, NP5-DM-COF (10 mg) and copper acetate monohydrate (0.033 mmol, 6.6 mg) were dissolved in 2 mL of ethanol. The ethanol solution (2 mL) of trimesic acid (0.016 mmol, 3.4 mg) was added to the above mixture. Then, add 0.2 mL of glacial acetic acid (17 M) to the suspension. The mixture was stirred at room temperature for 12 hours. Finally, the mixture was filtered and washed three times with ethanol and acetone to obtain a dark brown powder. The mixture was dried in an oven at 50 °C for 12 hours.

**Section 3. Photocatalytic performance and H_2_S-responsiveness of COF/HKUST-10**

**3.1 Total reactive oxygen species (ROS) generation**

To measure total ROS using DCFH-DA as a probe, NP5-DM-COF (500 μg·mL^-1^), COF/HKUST-10 (600 μg·mL^-1^), and H_2_O_2_ (1 mM) were dispersed in PBS (pH=7.4) according to the experiment requirements. DCFH-DA (2 μM) was then added to the mixture. Following incubation under different conditions (dark conditions or blue light (440 nm LED, 100 mW cm^-2^)), fluorescence signals (488/525 nm) were collected using a microplate reader.

**3.2 Detection of ^1^O_2_ generation**

The extracellular ROS generated by COF/HKUST-10 under blue light irradiation was characterized using the ^1^O_2_ probe DPBF. Blue light was continuously applied until the characteristic absorption peak of DPBF at 415 nm nearly disappeared. The absorbance of DPBF at 415nm was then measured with a microplate reader.

**3.3 Detection of** **O_2_^•−^ generation**

The production of O_2_^•−^ by COF/HKUST-10 was detected using the NBT reduction method. The absorbance changes were then measured using a UV-Vis spectrophotometer.

**3.4 Detection of •OH generation**

•OH effectively catalyzes the conversion of TMB in an acetic acid buffer solution, resulting in a distinct blue color with a characteristic absorption peak at 652 nm. To assess •OH generation, the TMB solution (2 μL, 10 mg·mL^-1^ in dimethyl sulfoxide) was added to NP5-DM-COF (500 μg·mL^-1^, pH = 4.5) and COF/HKUST-10 (600 μg·mL^-1^, pH = 4.5) acetic acid buffer solutions containing H_2_O_2_ (1 mM). Absorbance was then measured at OD_652_ using a microplate reader under blue light for a predetermined time. The effects of light exposure, varying concentrations of COF/HKUST-10, and different concentrations of H_2_O_2_ on •OH generation were evaluated using TMB detection.

**3.5 Photodegradation of MB**

The photocatalytic performance of the material was validated through a MB degradation experiment. NP5-DM-COF (500 μg·mL^-1^), COF/HKUST-10 (600 μg·mL^-1^), and H_2_O_2_ (1 mM) were mixed with a MB solution (10 μg·mL^-1^) according to experimental requirements, and stirred in the dark for 30 minutes to achieve adsorption-desorption equilibrium. Under blue light irradiation, the absorbance of MB at OD_664_ was recorded every 5 minutes using a microplate reader.

**3.6 Detection of ROS species**

In the presence of COF/HKUST-10, EPR signals were recorded under both dark and light irradiation conditions. DMPO and TEMP were added as trapping agents to detect O_2_^•−^ and ^1^O_2_, respectively. Additionally, H_2_O_2_ was added to COF/HKUST-10, with DMPO serving as a collector for •OH.

**3.7 H_2_S-responsiveness of COF/HKUST-10**

To study H_2_S responsiveness, NaHS (3 mM) was introduced into a COF/HKUST-10 solution to simulate endogenous H_2_S. Meanwhile, the reaction process was monitored using UV-Vis-NIR absorption spectroscopy. The absorbance changes in the NIR-II region were measured for reactions between NaHS (3 mM, physiological concentration) and different concentrations of COF/HKUST-10 (0.5, 1, 2, 3 mg·mL^-1^). Additionally, the time-dependent absorbance change was measured for the reaction between NaHS (3 mM, physiological concentration) and COF/HKUST-10 (2 mg·mL^-1^) at a fixed concentration.

**3.8 Detection of Cu^+^**

Neocuproine (1 mM) was selected as an indicator to detect in situ Cu^+^ production. COF/HKUST-10 (600 μg·mL^-1^) and NaHS (1 mM) were mixed with the indicator. After 10 minutes of light irradiation, the absorbance of the reaction solution was measured using a microplate reader.

**Section 4. *In vitro* antibacterial experiment**

**4.1 Bacterial cultivation and biofilm formation**

The Institutional Review Board of Jilin University, School of Dentistry, approved the selection of *P. gingivalis*, *F. nucleatum*, and *S. gordonii* as bacterial models for periodontal disease. These bacteria were cultivated under anaerobic conditions (80% N_2_, 10% H_2_, and 10% CO_2_) in a conditional medium of Tryptic Soy Broth (TSB) supplemented with menadione (1 mg·L^-1^), L-cysteine hydrochloride (0.5 g·L^-1^), yeast extract (5 g·L^-1^), and hemin (5 mg·L^-1^).

To establish a cohesive biofilm of *P. gingivalis* and *F. nucleatum*, the bacteria were inoculated onto cover glasses in a 24-well plate at a concentration of 10^8^ colony-forming units (CFUs)·mL^-1^. For the formation of a multispecies biofilm, a defined bacterial community containing *S. gordonii* (10^7^ CFUs·mL^-1^), *F. nucleatum* (10^7^ CFUs·mL^-1^), and *P. gingivalis* (10^7^ CFUs·mL^-1^) was introduced onto cover glasses in a 24-well plate. All biofilms were cultured in TSB-supplemented conditional medium at 37°C for 96 hours, with the medium replaced every 24 hours. This four-day incubation period allowed for the formation of mature periodontal biofilm.

**4.2 Detection of H_2_S produced by periodontopathic bacteria**

Periodontopathic bacteria metabolize sulfur-containing compounds, releasing H_2_S. H_2_S reacts with lead acetate (Pb(CH₃COO)_2_) on lead acetate test paper to form black lead sulfide (PbS), causing the paper to darken. This reaction is used to detect a bacterium’s ability to produce H_2_S. A bacterial suspension was adjusted to a concentration of 1×10⁸ CFU mL^-1^. A strip of lead acetate test paper was secured at the mouth of the test tube, ensuring no direct contact with the culture medium. The test tube was then sealed and incubated at 37 °C for 24 hours.

Reaction equation between H_2_S and Pb(CH₃COO)_2_:

H_2_S + Pb(CH_3_COO)_2_→PbS↓ + 2CH_3_COOH

**4.3 ROS detection of biofilms**

The DCFH-DA ROS probe was used to qualitatively assess ROS levels within the biofilm matrix. After rinsing the mature biofilm with sterile PBS, the biofilm was treated according to the designated experimental groups (PBS, NP5-DM-COF + L, COF/HKUST-10, COF/HKUST-10 + H_2_O_2_, COF/HKUST-10 + L, COF/HKUST-10 + H_2_O_2_ + L). Subsequently, the supernatant was discarded, and the bacterial biofilm was gently washed once with PBS. The DCFH-DA probe was added and allowed to specifically label ROS during a 30-minute incubation at 37℃. Excess probe was then removed by rinsing with PBS. Finally, the fluorescence intensity was subsequently analyzed using fluorescence microscopy and quantified with Image J.

**4.4 Live/dead bacterial viability assay**

The biofilm was stained with a mixture of SYTO9 and propidium iodide (Live/Dead Baclight Bacterial Viability Kit) to analyze live/dead bacteria. Biofilm visualization was conducted using confocal laser scanning microscopy (CLSM). Live and dead bacteria were quantified using Image J, and the mortality rates were calculated.

**4.5 Bacterial SEM**

Bacterial samples were collected in 1.5 mL Eppendorf tubes and centrifuged at 6000 rpm for 10 minutes to obtain bacterial pellets. The pellets were washed three times with PBS, followed by centrifugation to remove the supernatant. Fixation was performed using 2.5% glutaraldehyde in PBS for four hours. Gradient dehydration was carried out using a series of ethanol solutions with increasing concentrations from 30% to 100%, with each step lasting 10 minutes. Finally, the samples were placed on silicon wafers, sputter-coated with gold, and examined by SEM.

**4.6 CFU counts**

The bacterial biofilms were treated with COF/HKUST-10 at a concentration of 50 μg·mL⁻^1^, as well as with NP5-DM-COF at the corresponding concentration. They were then exposed to 440 nm LED light (100 mW cm^-2^) for 5 minutes, while the control group was kept in the dark. Biofilms were collected from the specimens by mechanical scraping. The resulting biofilm suspensions were serially diluted 10-fold and streaked onto Columbia blood agar plates. The plates were incubated at 37 °C in an atmosphere containing 5% CO_2_ for 24 hours for *F. nucleatum* and for 7 days for *P. gingivalis.* Following incubation, CFUs were counted, and CFU mL^-1^ were calculated based on the corresponding dilution factors.

**Section 5. Transcriptomic analysis.**

**5.1 RNA extraction and quality assessment of *P. gingivalis***

To investigate the antibacterial mechanism of COF/HKUST-10, *P. gingivalis* (ATCC 33277) was selected as a representative pathogen for transcriptomic analysis. The analysis compared a control group with a group treated with COF/HKUST-10 under light (COF/HKUST-10 + L). A *P. gingivalis* suspension (1×10^8^ CFU mL^-1^) was seeded into 24-well plates containing coverslips and allowed to form biofilms. Once established, the biofilms in the experimental group were treated with COF/HKUST-10 + L. After treatment, bacterial cells in the logarithmic growth phase were collected by washing the coverslips. The resulting cell suspensions were transferred to 1.5 mL nuclease-free tubes and centrifuged (10,000 rpm, 2 min). After repeated centrifugation steps to ensure removal of all supernatant, the bacterial pellets were snap-frozen in liquid nitrogen for 15 minutes, stored at −80°C, and subsequently shipped on dry ice for RNA extraction.

The construction of transcriptomic libraries and high-throughput sequencing (performed by Personalbio Co., Ltd.) followed this workflow: cDNA synthesis → cDNA library construction → Pre-sequencing preparation → Data filtering and quality control → Alignment with reference genome → Gene expression quantification.

**5.2 Principal component analysis (PCA) and differentially expressed genes (DEG) analysis**

Principal component analysis (PCA) was conducted using R software to reduce data dimensionality and visualize similarities among samples. Differential gene expression (DEGs) analysis was conducted using the DESeq2 package in R. Genes were considered differentially expressed if P < 0.05 and |log₂FoldChange| > log₂1.2.

**5.3 Enrichment analysis of differentially expressed genes**

The identified DEGs were subjected to Gene Ontology (GO) and Kyoto Encyclopedia of Genes and Genomes (KEGG) pathway enrichment analyses using the topGO package. DEGs were annotated with GO terms and KEGG pathways, and the number of genes associated with each term or pathway was quantified. The statistical significance of enrichment was then calculated.

**Section 6. *In vivo* antibacterial experiment**

**6.1 Ligation periodontitis rat model**

Male Wistar rats (~200 g) were used in this study, which was approved by the Ethics Committee of Medical Experiment Animals at the College of Basic Medicine of Jilin University (Changchun, China) under ethics approval number 2025 No. 436. Following a 1-week acclimation period, rats were anesthetized and ligature wires were placed around the maxillary second molars. The following day, *S. gordonii* was inoculated around the maxillary second molars, followed by *P. gingivalis* and *F. nucleatum* inoculations at 24-hour intervals. The ligature remained in place for 9 days to establish a periodontitis model. After successful establishment of the periodontal inflammation model, the ligatures were removed. The rats were then randomly assigned to the following treatment groups: Periodontitis group (PBS), Minocycline group, COF/HKUST-10 group, and COF/HKUST-10+L group. The subgingival nature of periodontitis necessitates a light source capable of penetrating soft tissue; herein, a commercial dental LED curing lamp was utilized. The gingival tissue in adult Wistar rats possesses a millimeter-scale thickness, which allows the blue light to effectively traverse the tissue and reach the subgingival biofilm (0.5-1.5 mm). Each group was administered 0.2 mL of the respective treatment in situ, followed by irradiation with a commercial clinical-standard dental curing light (420**–**480 nm, λ_peak_ = 447 nm, 50 mm^2^ spot area, 1000 mW·cm^-2^, 5 minutes, 300 J·cm^-2^, Great Life: WM-212-C) once daily for three consecutive days.

**6.2** **Antibacterial effect assessment *in vivo***

The therapeutic effect on periodontal inflammation was evaluated using the standard plate counting method. In brief, a sterile swab was brushed over the infected site for 10 seconds and then placed in a sterile tube. Culture medium was added to the tube, which was subsequently incubated at 37 ℃ for 48 hours. The suspensions were then serially diluted and cultured on blood agar plates.

**6.3 Microcomputed tomography (Micro-CT) evaluation**

Following treatment completion, the rats in each experimental group were euthanized, and the soft tissue from the treatment site was surgically removed. The samples were then fixed in 4% paraformaldehyde solution. The extracted maxillary samples were scanned using a μCT50 (Scanco Medical AG, Bassersdorf, Switzerland) at a resolution of 70 kVp and 200 μA, and analyzed using V6.5-3 analytical software. The distance between the cementoenamel junction (CEJ) and the alveolar bone crest (ABC) was measured to assess linear alveolar bone loss.

**6.4 Histological evaluation**

The tissue was embedded in paraffin wax, followed by immunofluorescence staining, H&E staining, and Masson's trichrome staining to further assess the therapeutic efficacy.

**Section 7. Biosafety assay of NP5-DM-COF-TA**

**7.1 The cell viability**

L929 mouse fibroblasts were cultured in DMEM supplemented with 10% fetal bovine serum (FBS) and 1% antibiotics, and incubated at 37 ℃ under 5% CO_2_. A 100 μL cell suspension containing L929 cells was seeded into each well of a 96-well plate at a density of 3 × 10^3^ cells/well. After culturing for 24 hours, the cells adhered to the surface of the wells, and the supernatant was discarded. Subsequently, different concentrations of COF/HKUST-10 dissolved in culture medium (100 μL) were added to each well. For the light-exposed group, illumination at an intensity of 100 mW·cm^−2^ was administered for 5 minutes per day, starting one hour after drug-addition. Incubation continued at 37 ℃ for an additional 24 hours. Finally, cell viability was assessed using the CCK-8 method.

**7.2 The cell hemolysis assay**

Six-week-old healthy male Wistar rats (~250 g) were obtained from the Experimental Animal Center of Jilin University. Fresh blood samples were collected via abdominal aorta puncture and centrifuged at 1500 rpm for 15 minutes to isolate red blood cells. The isolated red blood cells were then washed with PBS until the supernatant was clear. A 4% w/v erythrocyte suspension and a sample with double the concentration were prepared. For the positive control (+), 0.5% Triton X-100 was utilized, while PBS alone served as the negative control (-). An equal volume of red blood cell suspension was combined with the sample solution and incubated at 37 ℃ for 2 hours. Subsequently, the blood mixture was centrifuged at 1500 rpm for 15 minutes, and the resulting supernatant was transferred to a 96-well plate for detection of OD values at a wavelength of 576 nm. The hemolysis rate was calculated according to the following formula:

$Hemolysis (\%)=\frac{OD sample -OD negative control}{OD positive control -OD negative control}$×100%

**Section 8. Statistical analysis**

The animals used for *in vivo* experiments were of the same age, and all *in vitro* studies represented multiple independent experiments conducted in triplicate. Data were presented as means ± standard deviation (SD) for at least three samples (n ≥ 3). Statistical analysis was performed using SPSS 24.0 software (SPSS, Chicago, IL, USA) employing Student's t-test and one-way analysis of variance (ANOVA), followed by a Tukey’s HSD post-hoc test. A significance level of p < 0.05 was considered statistically significant.

**Section 9. Supporting figures**

**
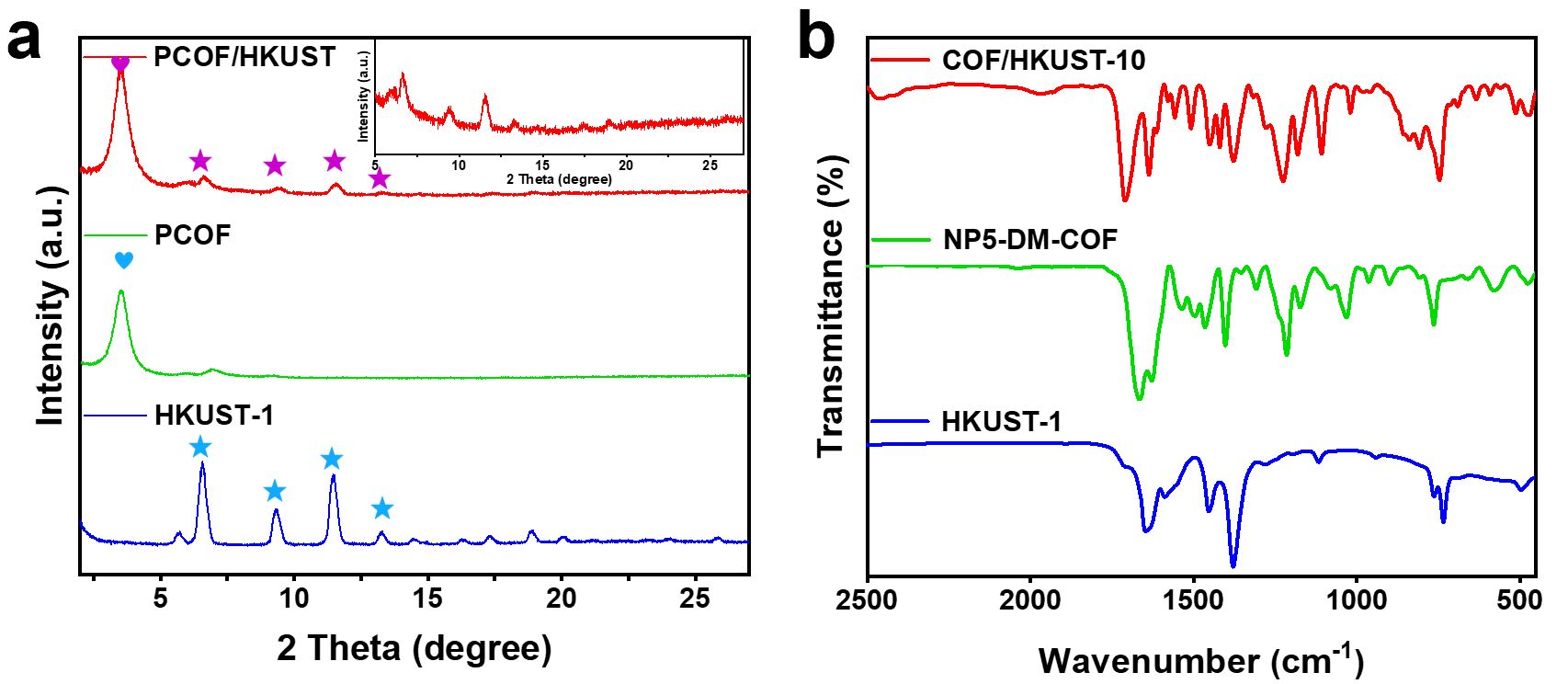
**

**Figure S1.** a) PXRD patterns of HKUST-1, NP5-DM-COF, and COF/HKUST-10. b) FTIR spectra of HKUST-1, NP5-DM-COF, and COF/HKUST-10.


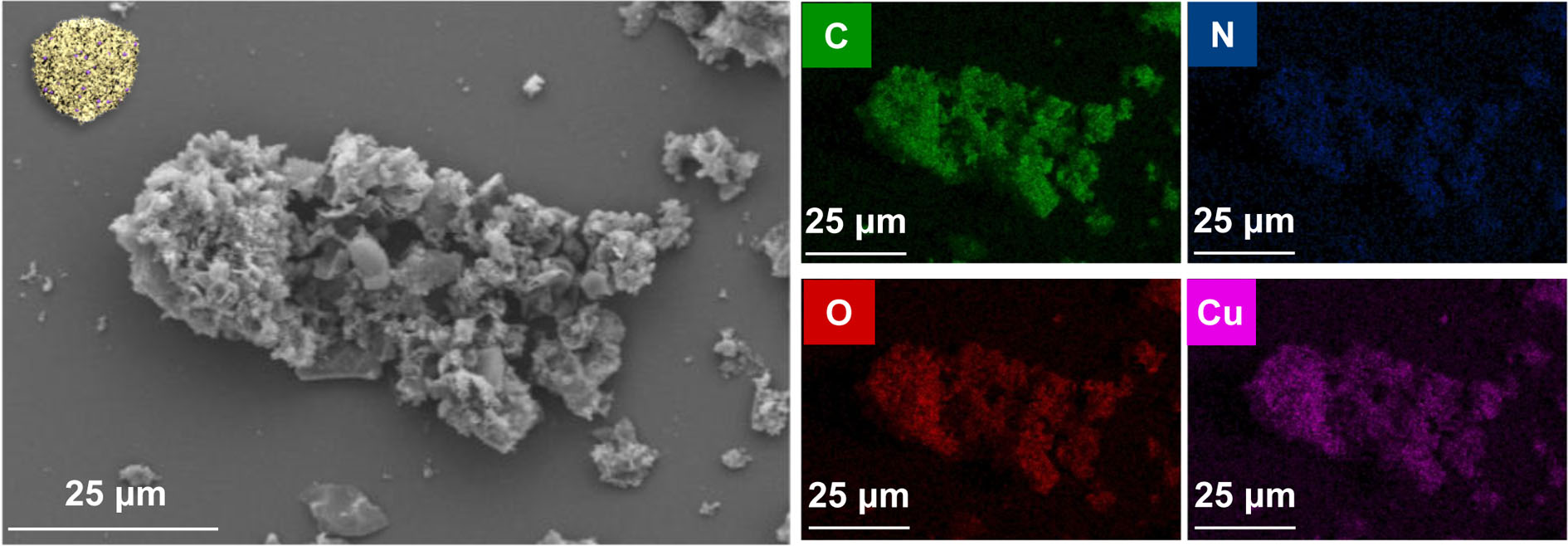


**Figure S2.** The SEM image and elemental mappings of C, N, O, and Cu in COF/HKUST-10.


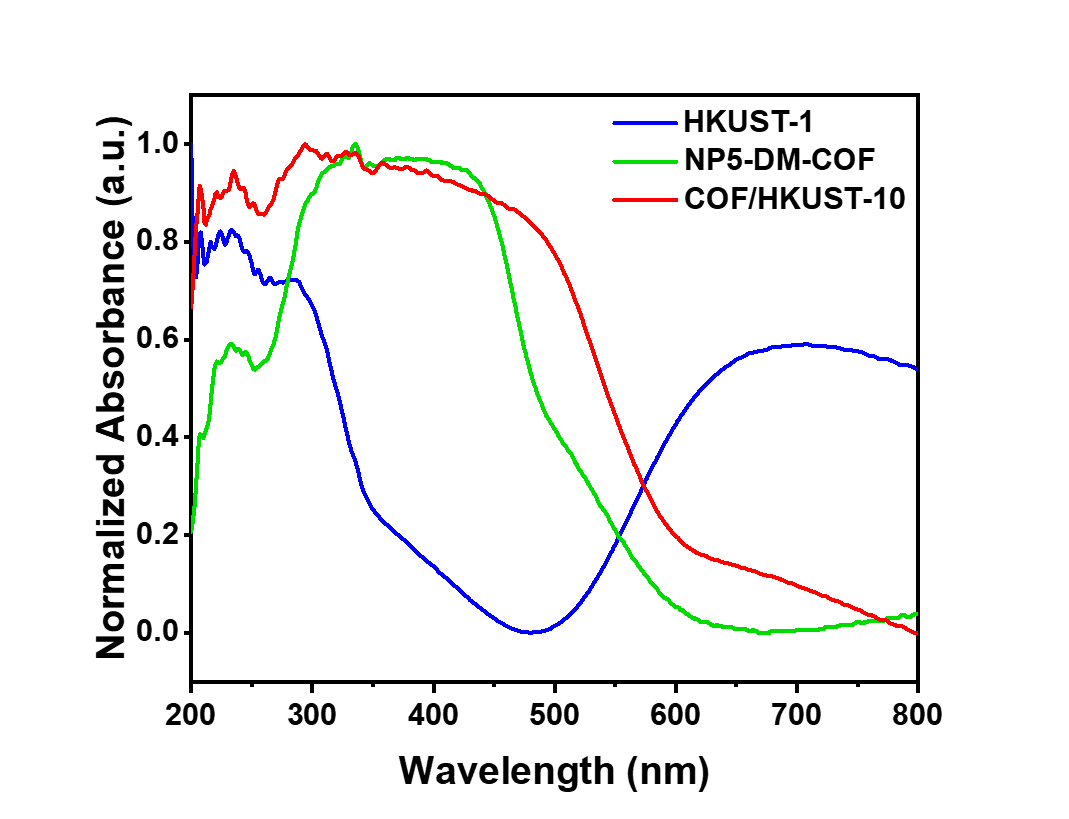


**Figure S3.** UV−vis−DRS spectra of HKUST-1, NP5-DM-COF, and COF/HKUST-10.


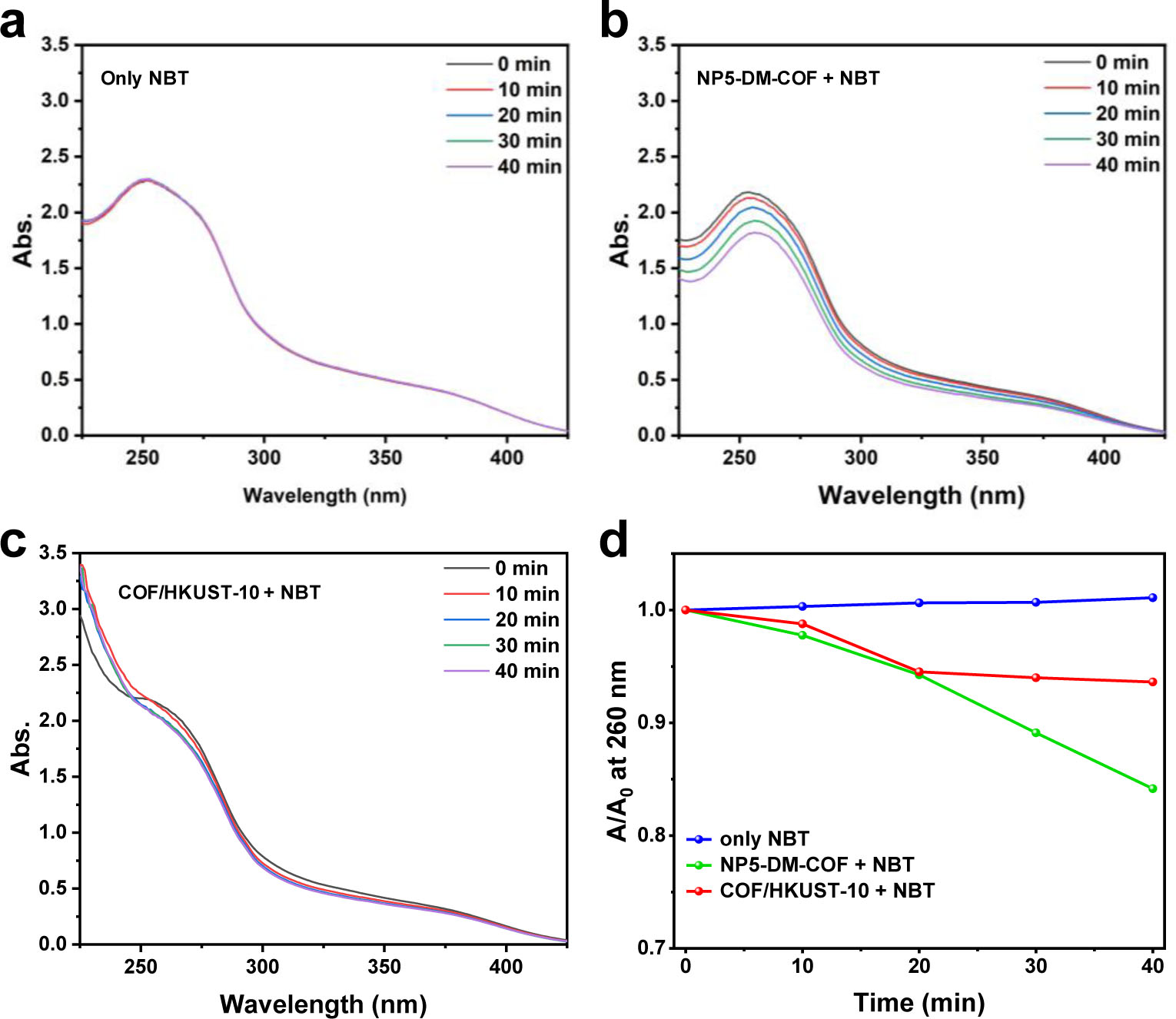


**Figure S4**. The UV-Vis absorption spectra of a) NBT + L alone, b) NP5-DM-COF + NBT + L, and c) COF/HKUST-10 + NBT + L at different time intervals. L stands for light irradiation. d) Quantification of the O_2_^•−^ generation capabilities of NP5-DM-COF and COF/HKUST-10.

**
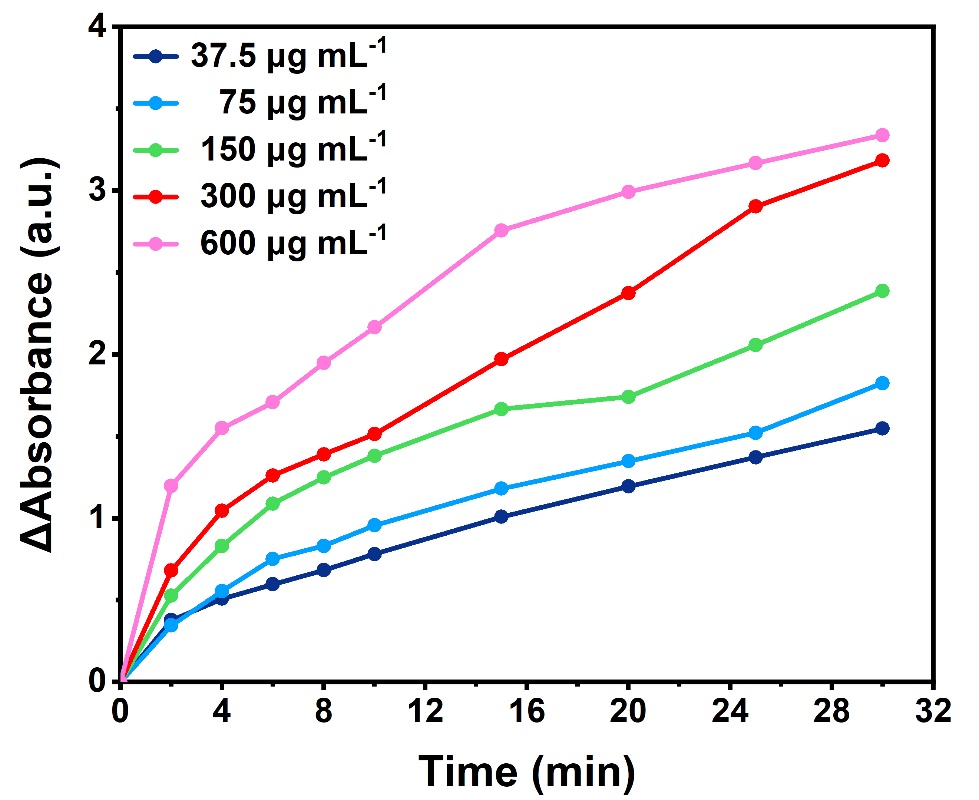
**

**Figure S5.** Concentration-dependent catalysis of H_2_O_2_ to •OH by COF/HKUST-10 (varying COF/HKUST-10 concentration).


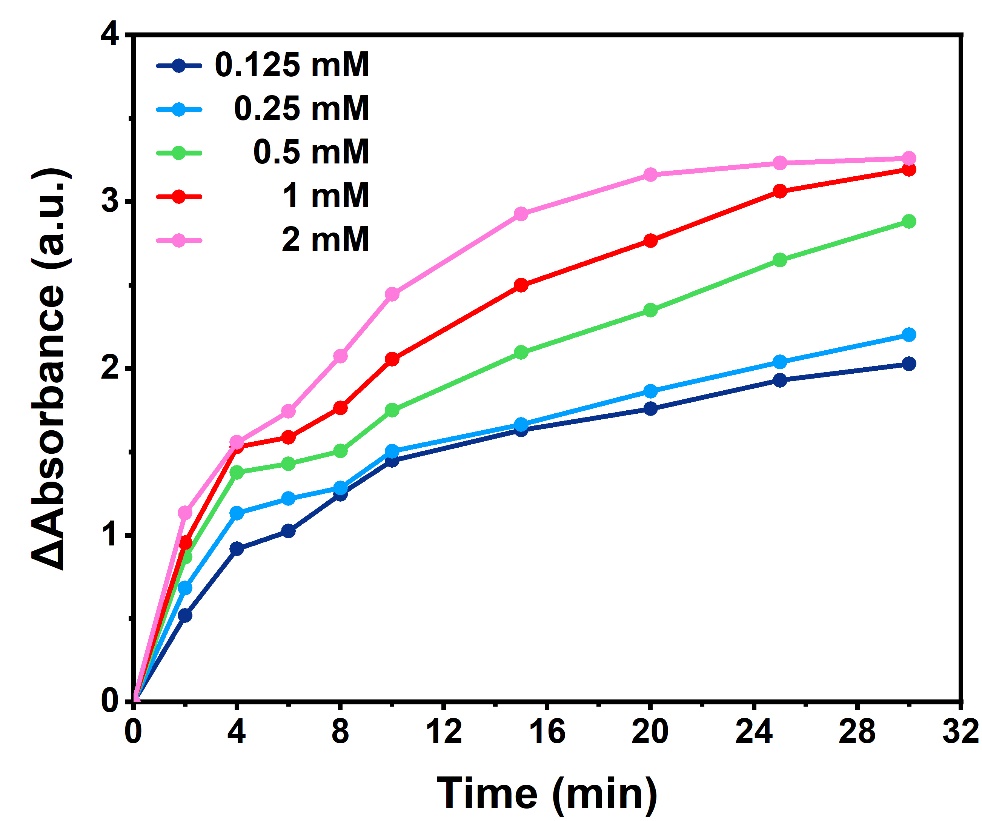


**Figure S6.** Concentration-dependent catalysis of H_2_O_2_ to •OH by COF/HKUST-10 (varying H_2_O_2_ concentration).

**Figure S7.** Quantification of •OH generation capabilities of NP5-DM-COF and COF/HKUST-10 under light irradiation.

**
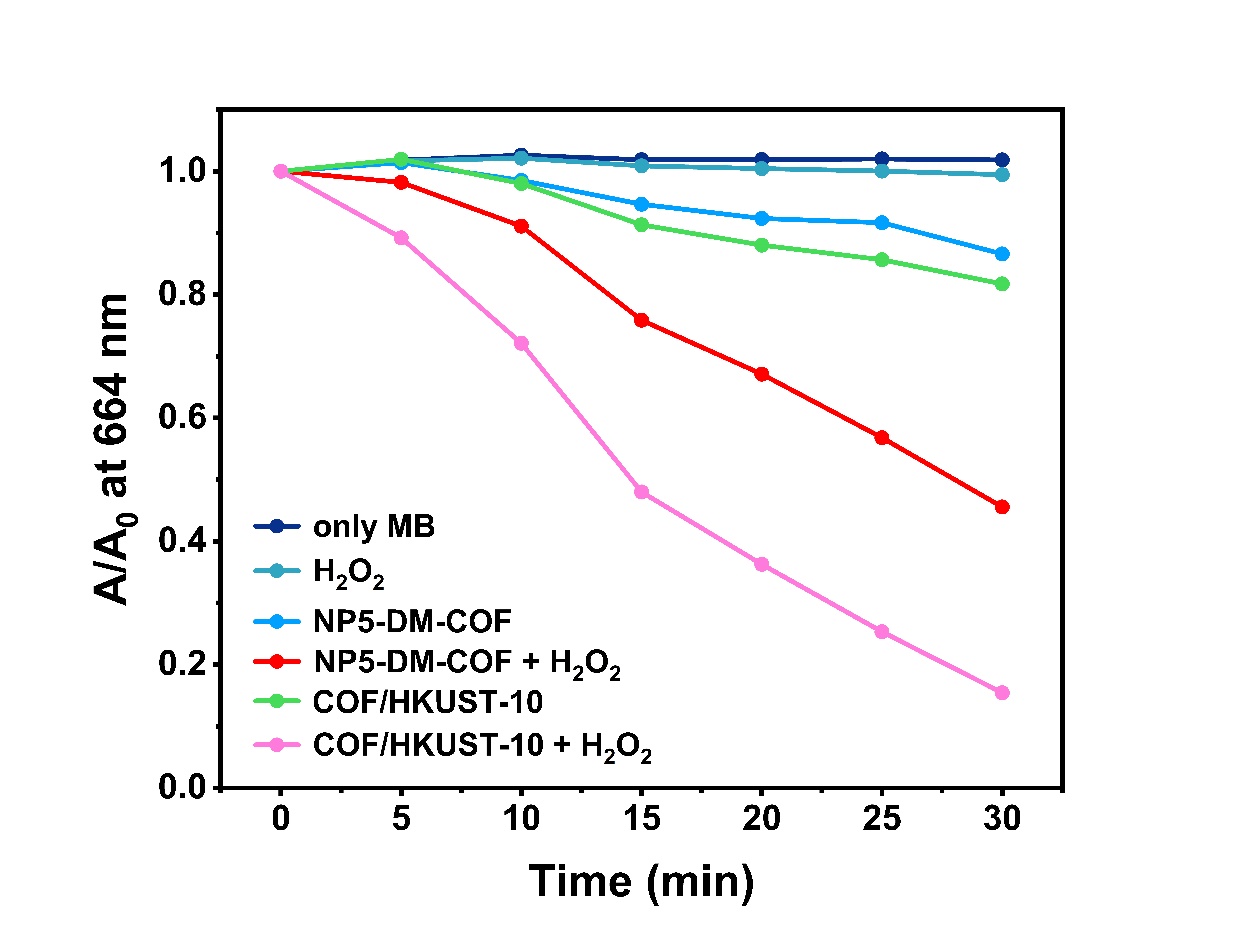
**

**Figure S8.** Degradation efficiency of MB photocatalyzed by the samples.

**
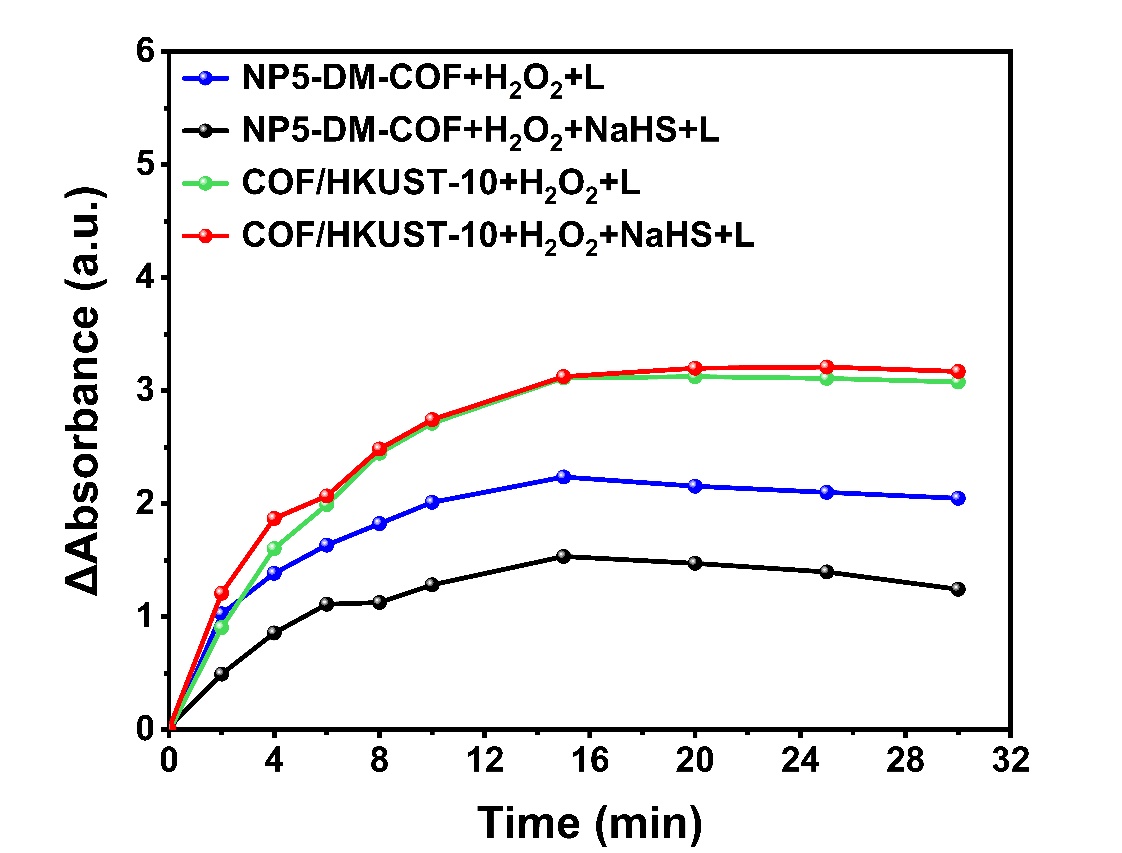
**

**Figure S9.** Effect of H_2_S on the production of ROS in NP5-DM-COF and COF/HKUST-10. L: light irradiation.


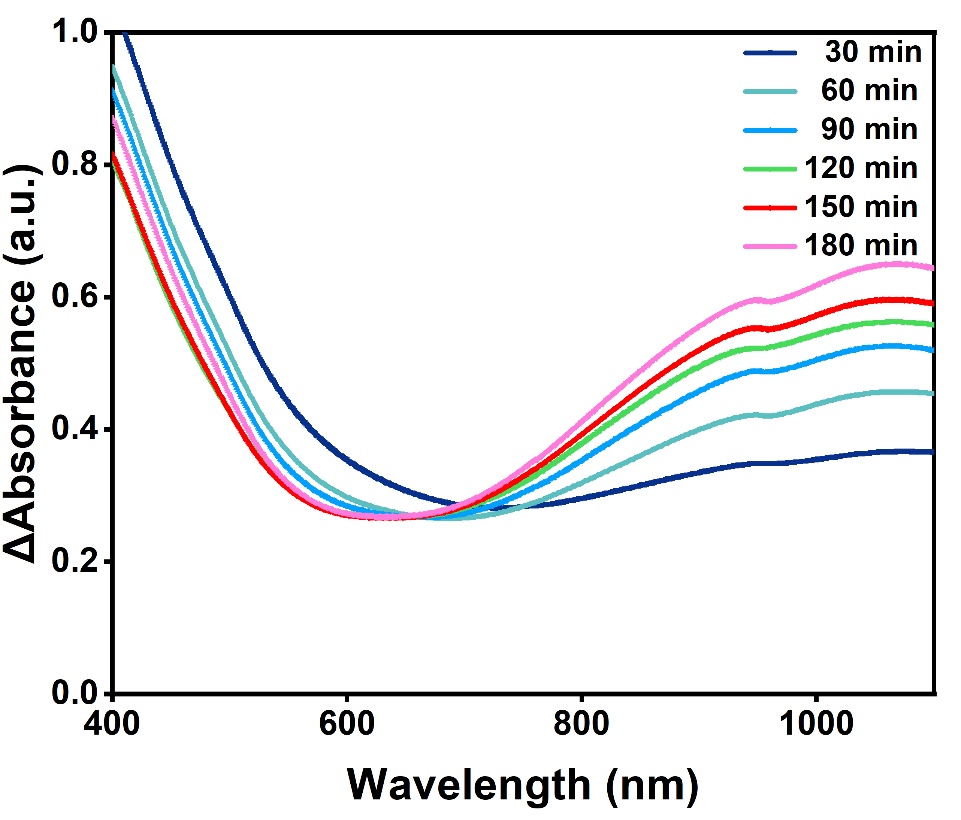


**Figure S10.** UV-vis-NIR spectra of H_2_S reacted with COF/HKUST-10 at different times.


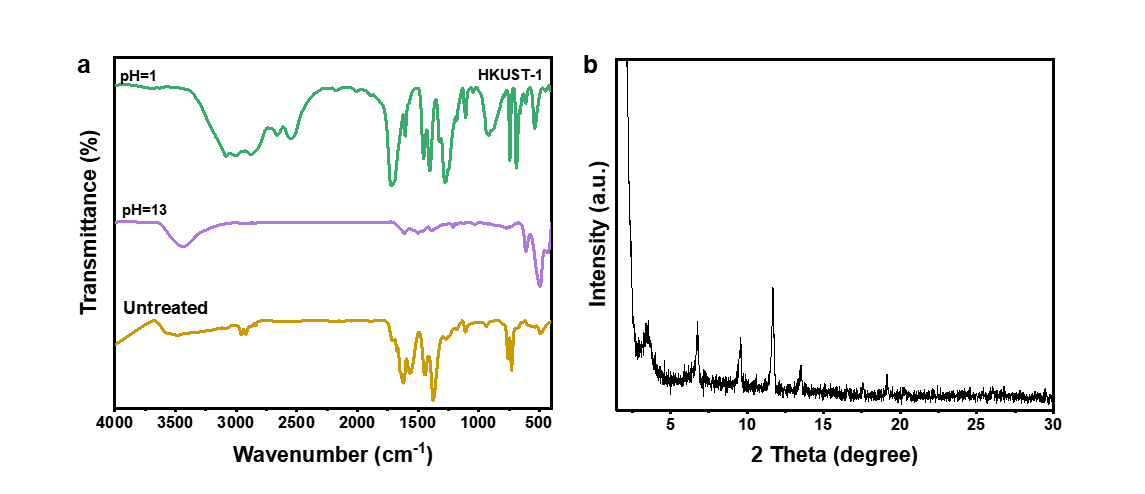


**Figure S11.** a) FTIR spectra of HKUST-1 in acid and base conditions. b) PXRD pattern of COF/HKUST-10 after 12 h immersion in artificial saliva.


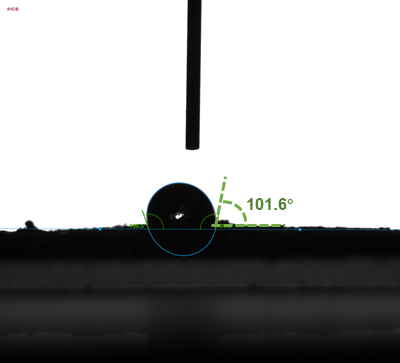


**Figure S12.** Water contact angle of NP5-DM-COF.


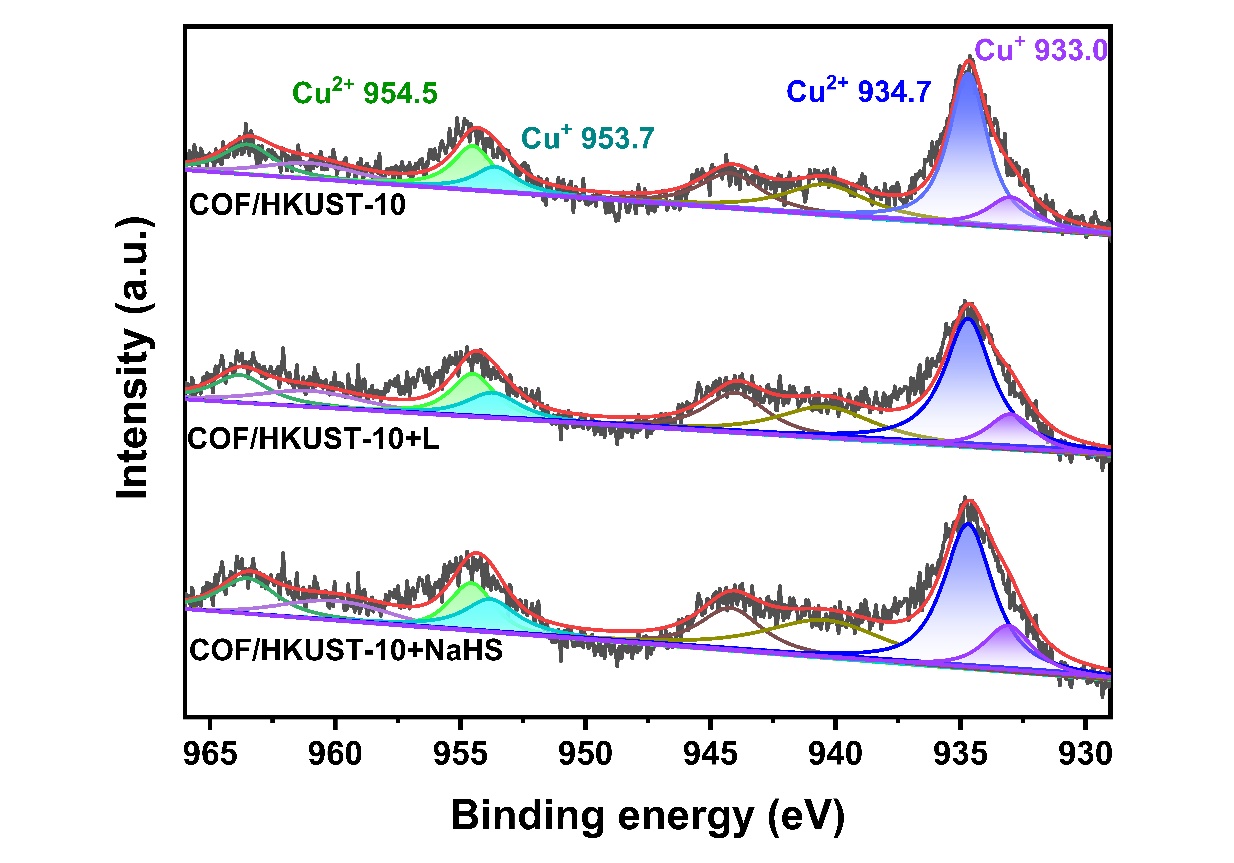


**Figure S13.** XPS spectra of COF/HKUST-10, COF/HKUST-10 + L, and COF/HKUST-10 + NaHS. L: light irradiation.

**
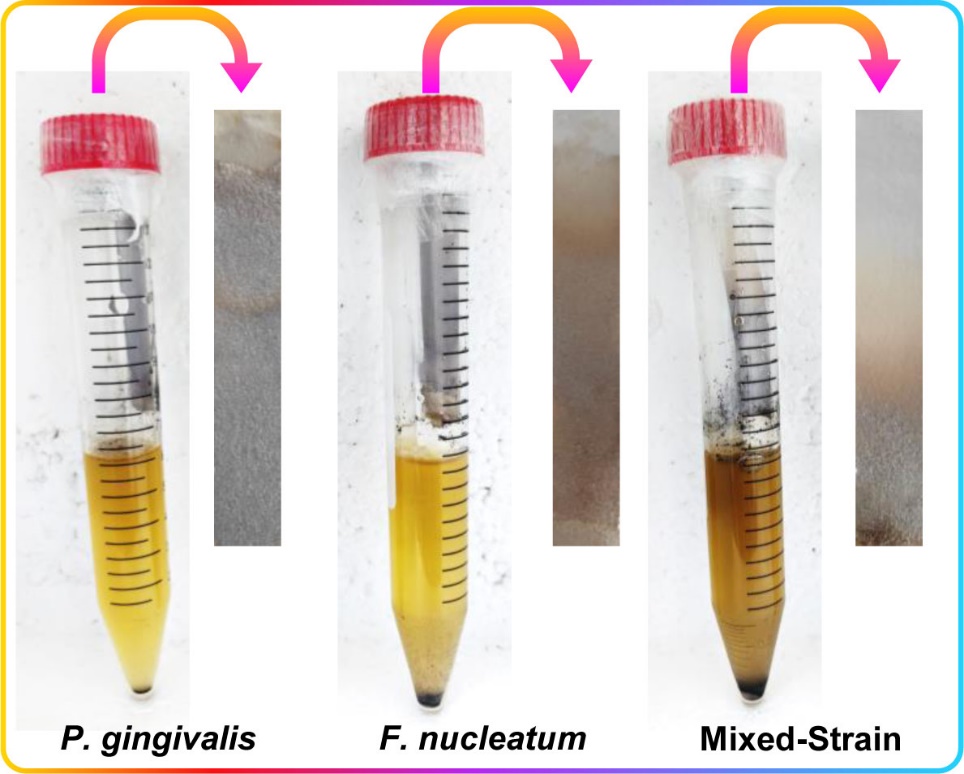
**

**Figure S14.** Detection of H_2_S gas produced by periodontal pathogens using lead acetate test paper.

**
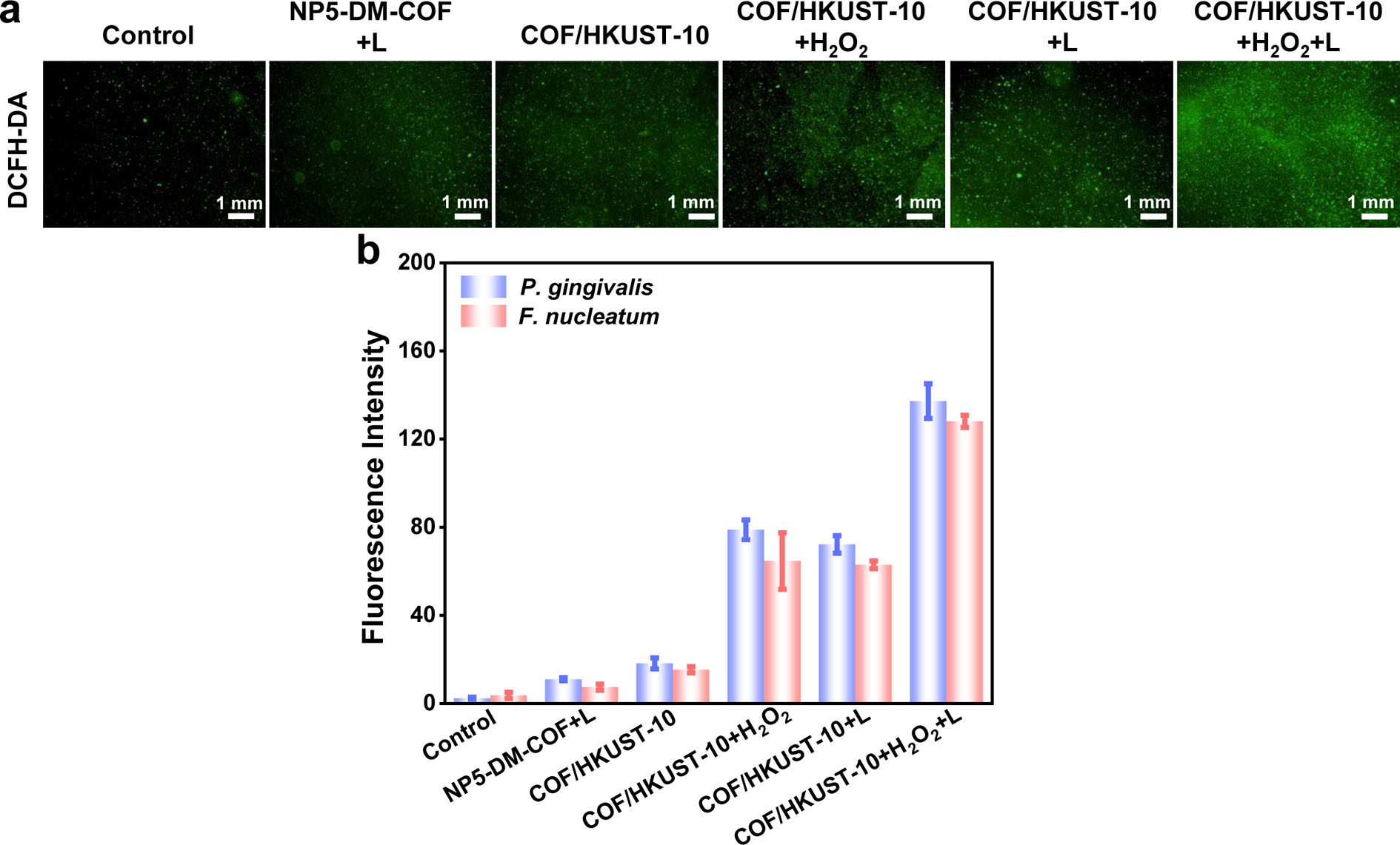
**

**Figure S15.** a) Fluorescence images showing ROS generation in *F. nucleatum* biofilms detected using DCFH-DA probe under various treatment conditions. b) The fluorescence intensity showing ROS generation in bacteria under various treatment conditions. L: light irradiation.


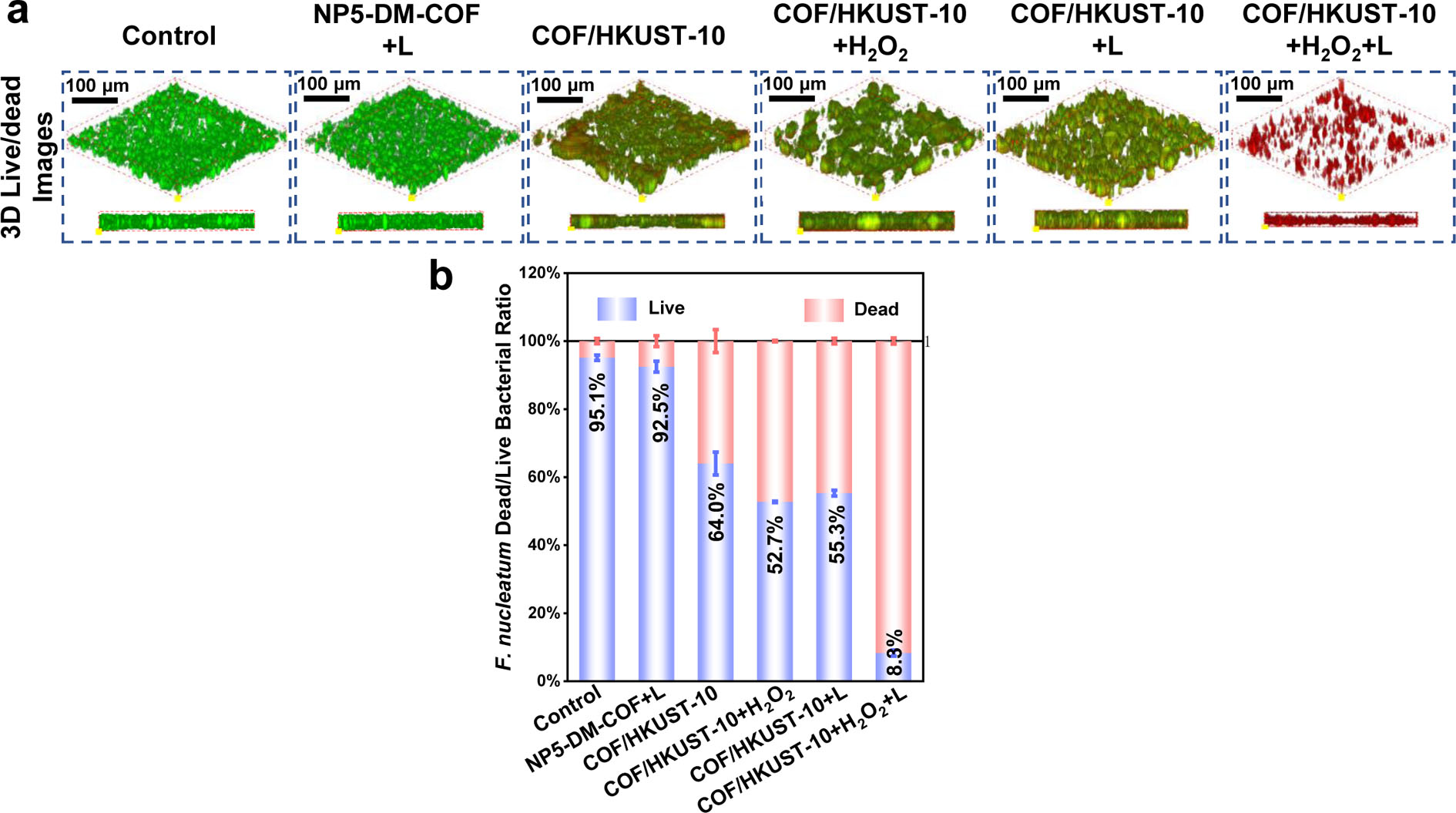


**Figure S16**. a) Representative 3D live/dead images showcasing *F. nucleatum* biofilms under different treatments. b) Live/dead bacteria ratio of *F. nucleatum* biofilms. L: light irradiation.


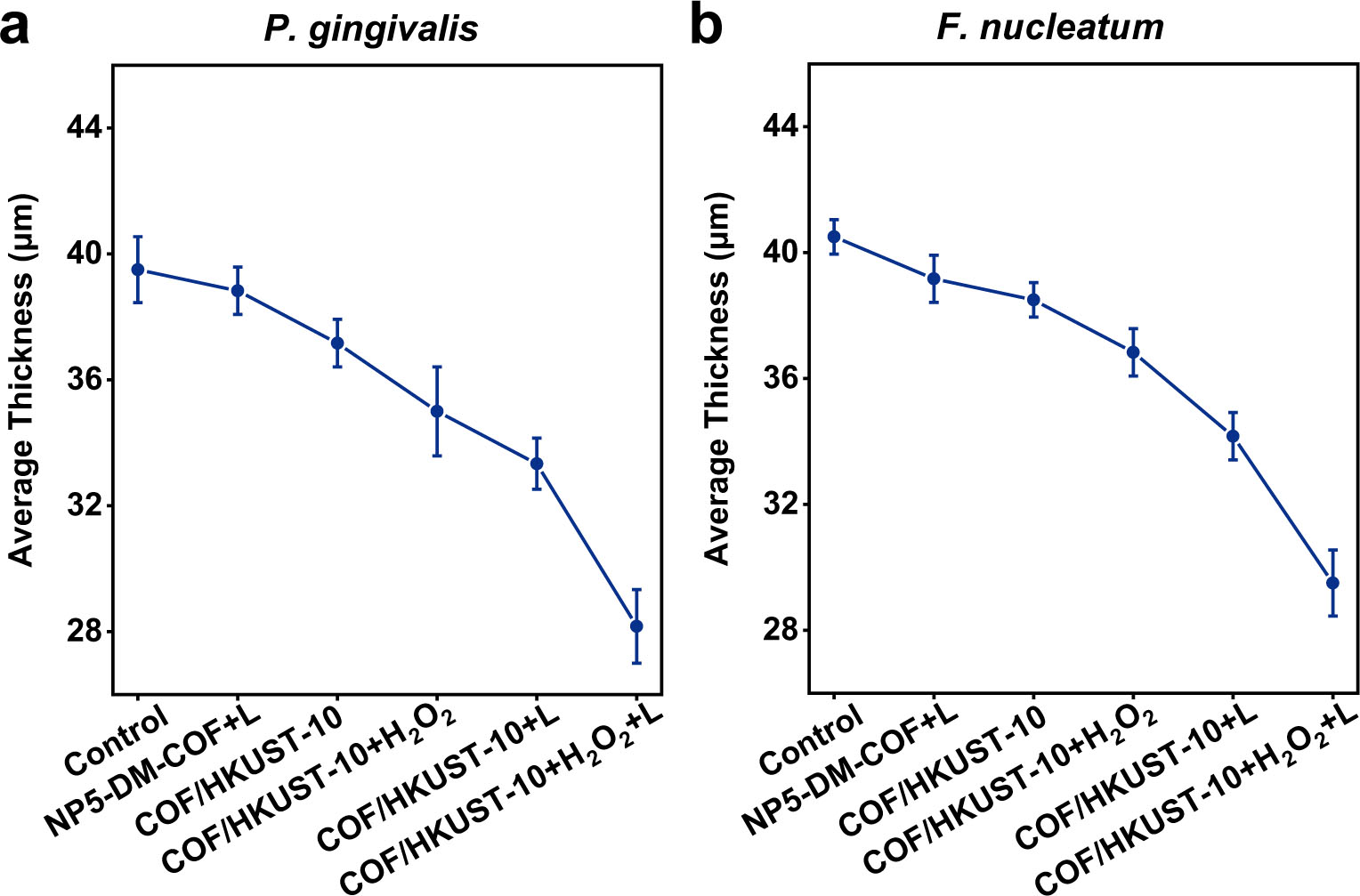


**Figure S17.** The average thickness of a) *P. gingivalis* and b) *F. nucleatum* biofilms. L means light irradiation.


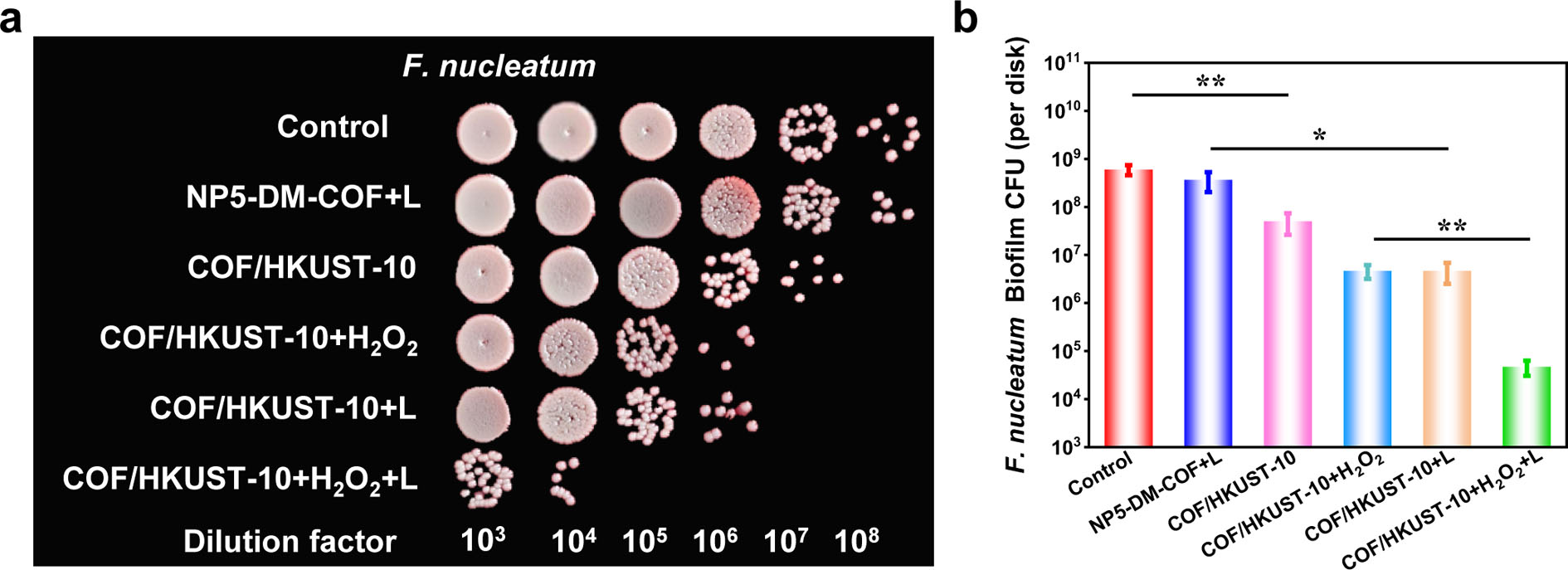


**Figure S18.** a) CFU count images of *F. nucleatum* biofilms. b) Corresponding statistical data. L: light irradiation.


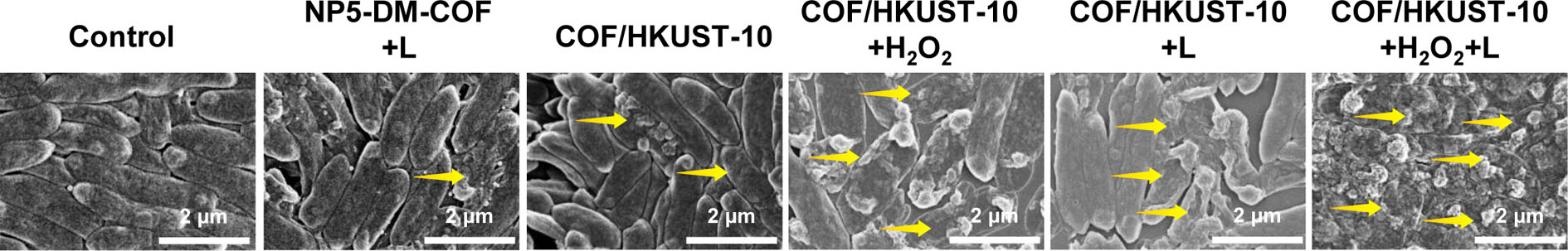


**Figure S19.** SEM images of *F. nucleatum* under different treatments. The yellow arrows indicate the characteristic morphology of damaged bacteria. L: light irradiation.


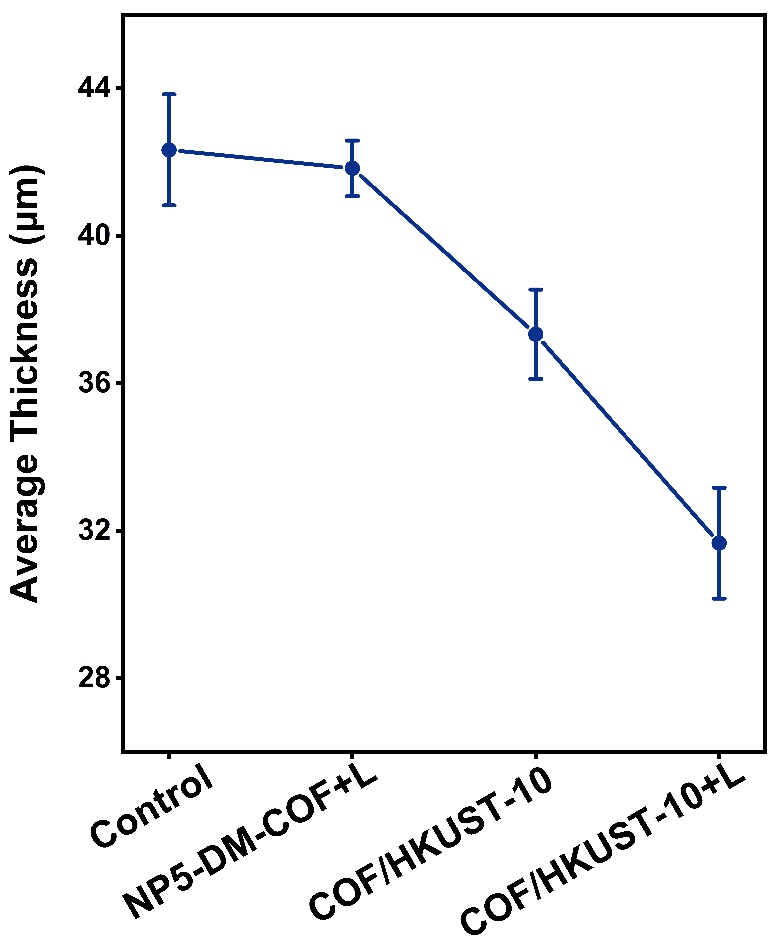


**Figure S20.** The average thickness of mixed-strain biofilms. L: light irradiation.


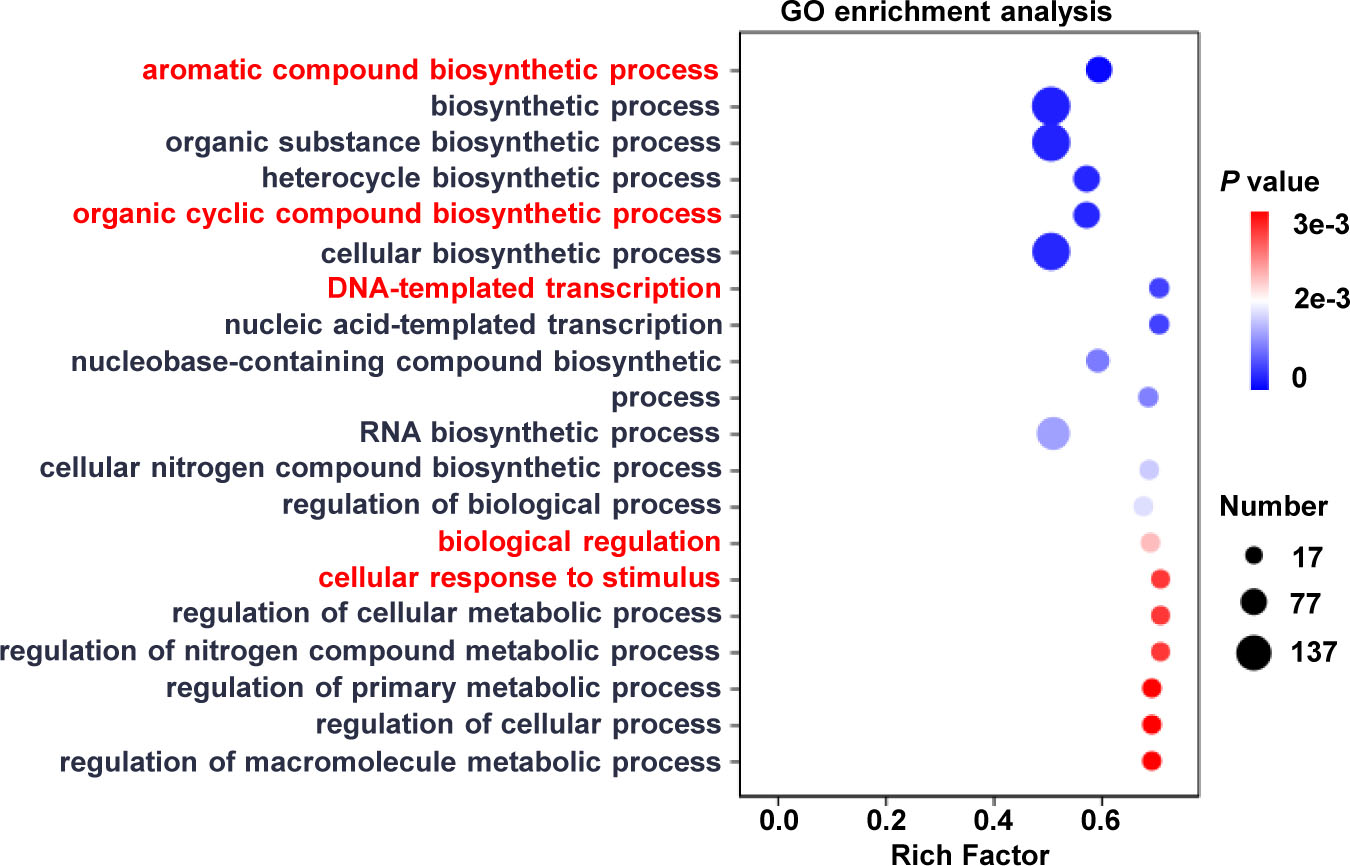


**Figure S21**. GO enrichment of top 20 relevant pathways in response to COF/HKUST-10 + L.


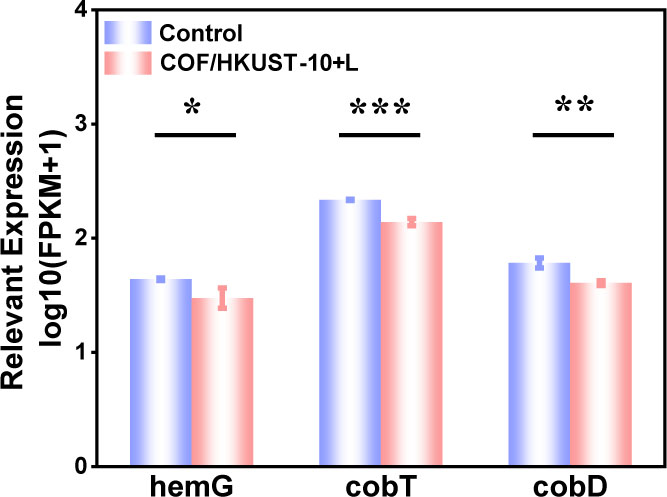


**Figure S22.** Quantitative analysis of genes involved in porphyrin metabolism. L means light irradiation.


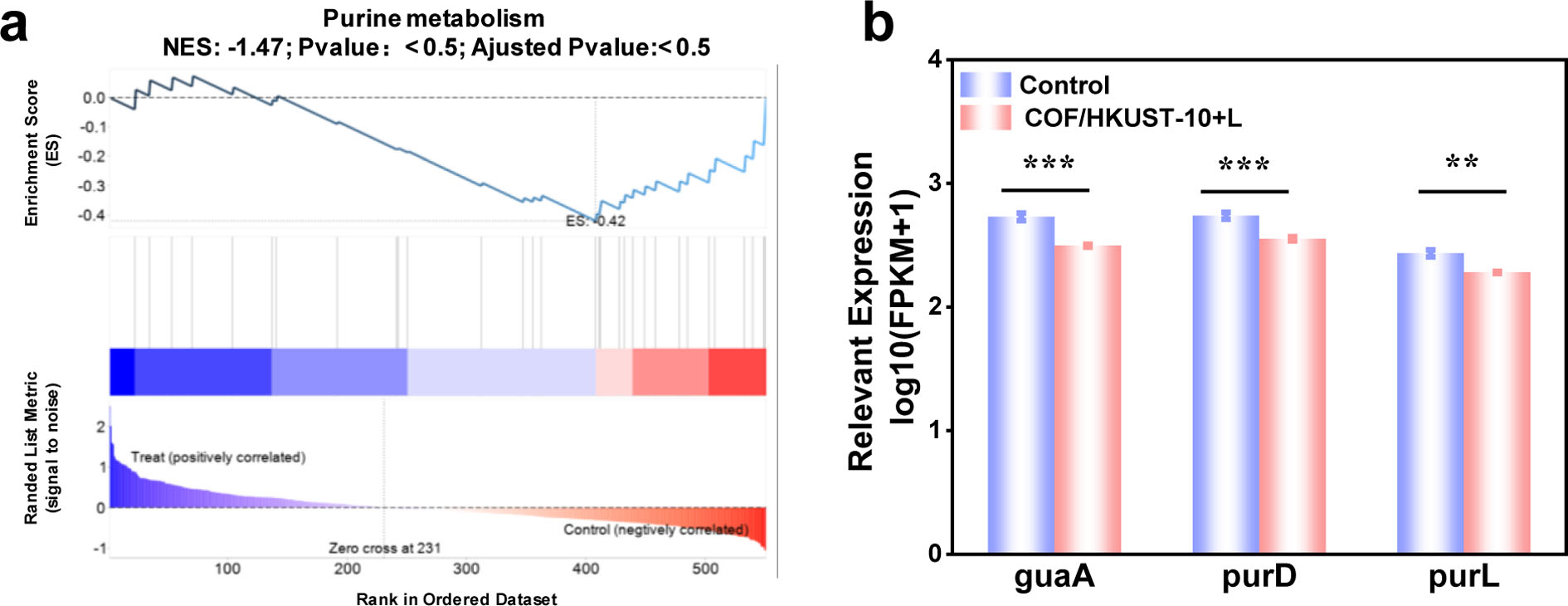


**Figure S23.** a) GSEA of the purine metabolism pathway. b) Quantitative analysis of genes related to purine metabolism. L: light irradiation.


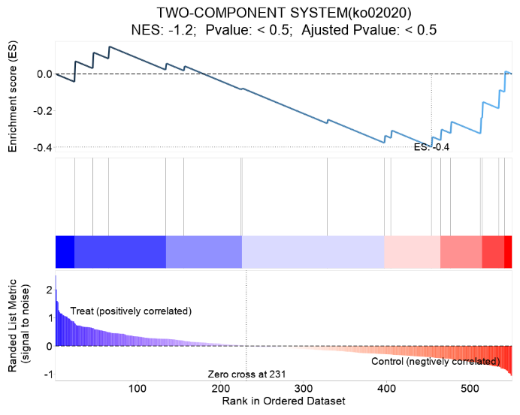


**Figure S24.** GSEA of two-component system.


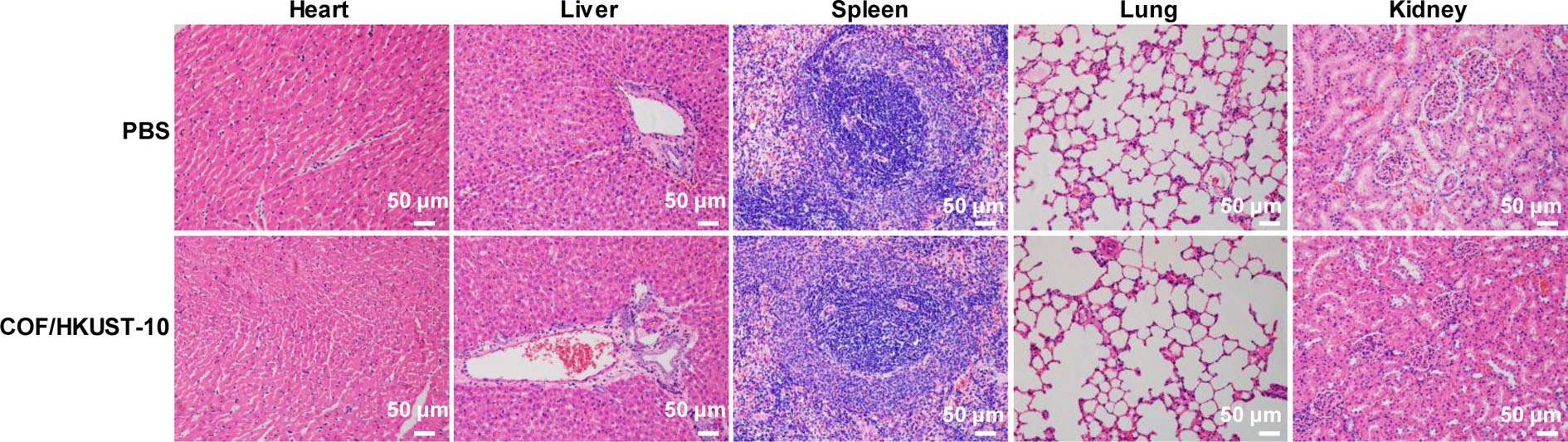


**Figure S25.** Histological images of main organs posttreatment for 7 days in Wistar rats.

**
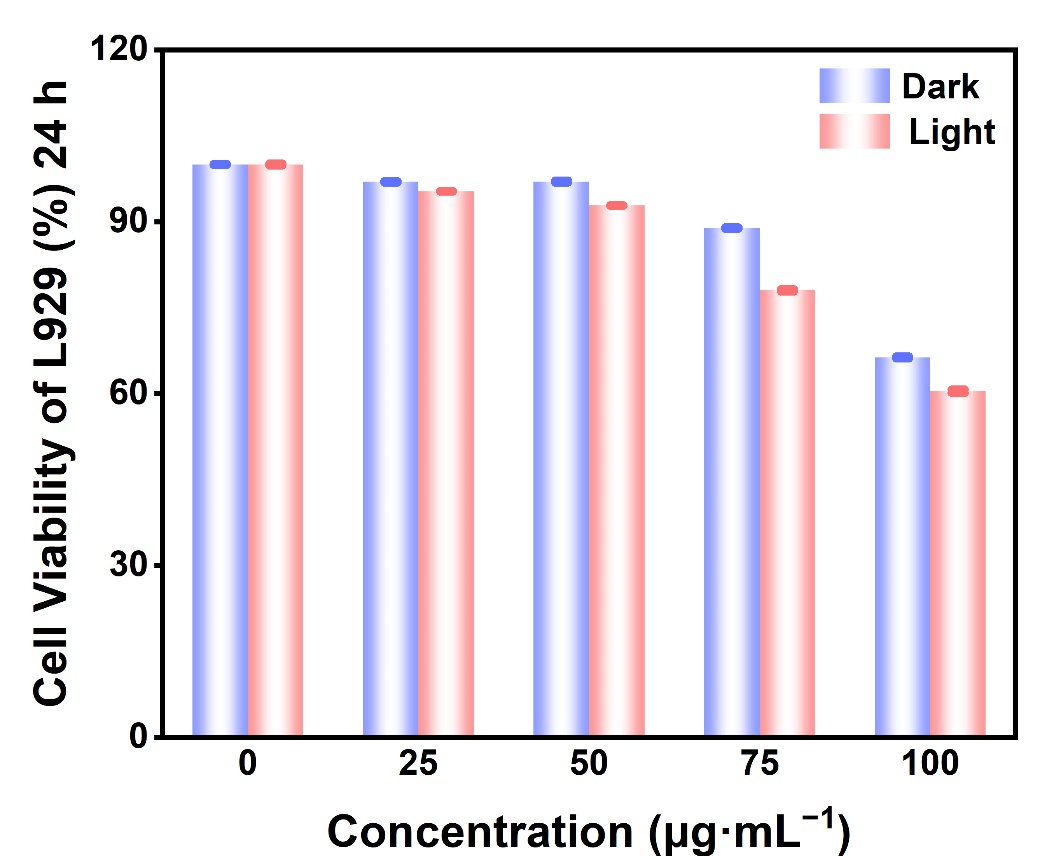
**

**Figure S26.** Effects of COF/HKUST-10 at varying concentrations on the viability of L929 cells.

**
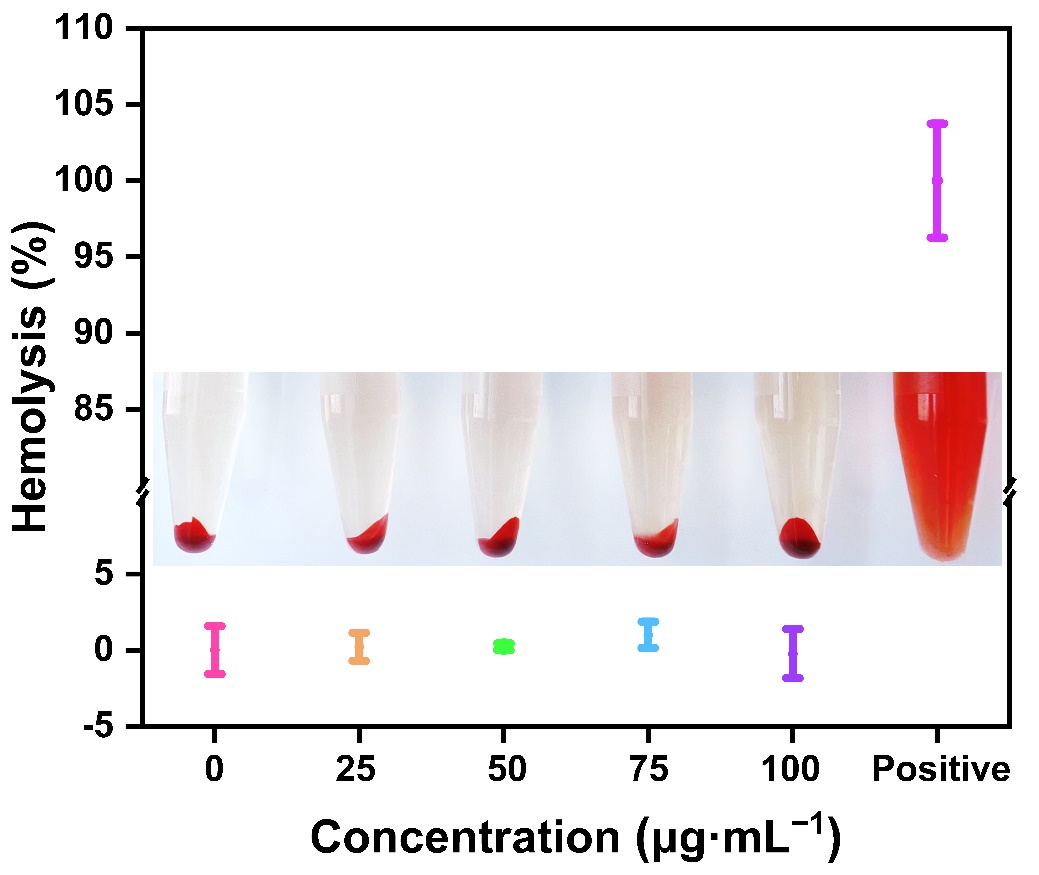
**

**Figure S27.** Hemolysis of COF/HKUST-10.

**References**

[S1] S. Liang, M.-H. Li, M.-L. Qi, et al., "Reactive Oxygen Species-Responsive Pillararene-Embedded Covalent Organic Frameworks with Amplified Antimicrobial Photodynamic Therapy for the Targeted Elimination of Periodontitis Pathogens," *Nano Letters* 24, no. 43 (2024): 13708.

[S2] M.-H. Li, Z. Yang, Z. Li, et al., "Construction of Hydrazone-Linked Macrocycle-Enriched Covalent Organic Frameworks for Highly Efficient Photocatalysis," *Chemistry of Materials* 34, no. 12 (2022): 5726.

[S3] M.-H. Li, Z. Yang, H. Hui, et al., "Superstructure-Induced Hierarchical Assemblies for Nanoconfined Photocatalysis," *Angewandte Chemie International Edition* 62, no. 47 (2023): e202313358.
